# Anti-angiogenic tyrosine kinase inhibitors and the pathophysiology of their toxic effects: revisiting the treatment of anemia in metastatic cancers

**DOI:** 10.1186/s40164-025-00640-9

**Published:** 2025-04-19

**Authors:** Tai Van Nguyen, Eurydice Angeli, Diaddin Hamdan, Morad El Bouchtaoui, Oanh T. Bui, Feriel Azibani, Rong Shen, He Lu, Kien Hung Do, Anne Janin, Quang Van Le, Guilhem Bousquet

**Affiliations:** 1 Université de Paris, INSERM, MASCOT, 75006 Paris, France; 2 Vietnam National Cancer Hospital – K Hospital, Hanoi, Vietnam; 3 Hanoi Medical University, Hanoi, Vietnam; 4 Université Sorbonne Paris Nord, 9 Avenue Jean Baptiste Clément, 93439 Villetaneuse, France; 5 Assistance Publique Hôpitaux de Paris, Hôpital Avicenne, Service d’oncologie Médicale, 93008 Bobigny, France; 6 Hôpital La Porte Verte, 78004 Versailles, France; 7 Department of Hematology, Shanghai Institute of Hematology, Ruijin Hospital Affiliated to Shanghai Jiao Tong University School of Medicine, No. 197, Ruijin 2Nd Road, Shanghai, 200025 China; 8 UMR_S942 Inserm-Université de Paris-Université Paris 13, UFR SMBH, 1 Rue Chablis, 93000 Bobigny, France

**Keywords:** Anti-angiogenic agents, Tyrosine kinase inhibitor, Anemia, Sunitinib, Autophagy, Erythropoietin

## Abstract

**Background:**

Anti-angiogenic tyrosine kinase inhibitors (TKIs) have become major drugs for the treatment of various cancer types, but with an overall high incidence of severe toxicities, particularly haematological toxicities including severe anemia.

**Methods:**

We treated C57BL6 mice continuously by gavage for 14 days with either sunitinib, pazopanib, or axitinib. In this study, we set out to decipher the pathophysiological mechanisms of anti-angiogenic TKI haematological toxicity.

**Results:**

We demonstrated that anti-angiogenic TKIs induced a broad range of toxic effects on normal tissues through a cytotoxic effect on normal endothelial cells. Haematological toxicities were particulary marked with sunitinib. Sunitinib-induced hypoxia through the destruction of normal vessels in the bone marrow mainly affected erythrocyte and myeloid lineages, and this was associated with a blockage in erythrocyte maturation. Althought sunitinib-induced anemia was associated with an adaptative response to systemic hypoxia, we demonstrated that erythropoietin (EPO) concentrations in the total bone marrow of sunitinib-treated mice were significantly lower than in untreated mice. This is coherent with the destruction of microvessels in the bone marrow under sunitinib treatment, preventing circulating EPO from reaching the bone marrow at relevant concentrations. However, we demonstrated an additional effect specific to sunitinib that induced autophagy flux inhibition in erythroid progenitors, with a blockage of erythrocyte maturation, leading to more severe anemia.

**Conclusions:**

We deciphered the pathophysiology of anti-angiogenic TKI-induced anemia, which we observed to be mainly linked to a direct effect on normal bone-marrow vessels and to autophagy flux inhibition in erythroid progenitors under sunitinib.

**Supplementary Information:**

The online version contains supplementary material available at 10.1186/s40164-025-00640-9.

## To the editor,

**Fig. 1:**
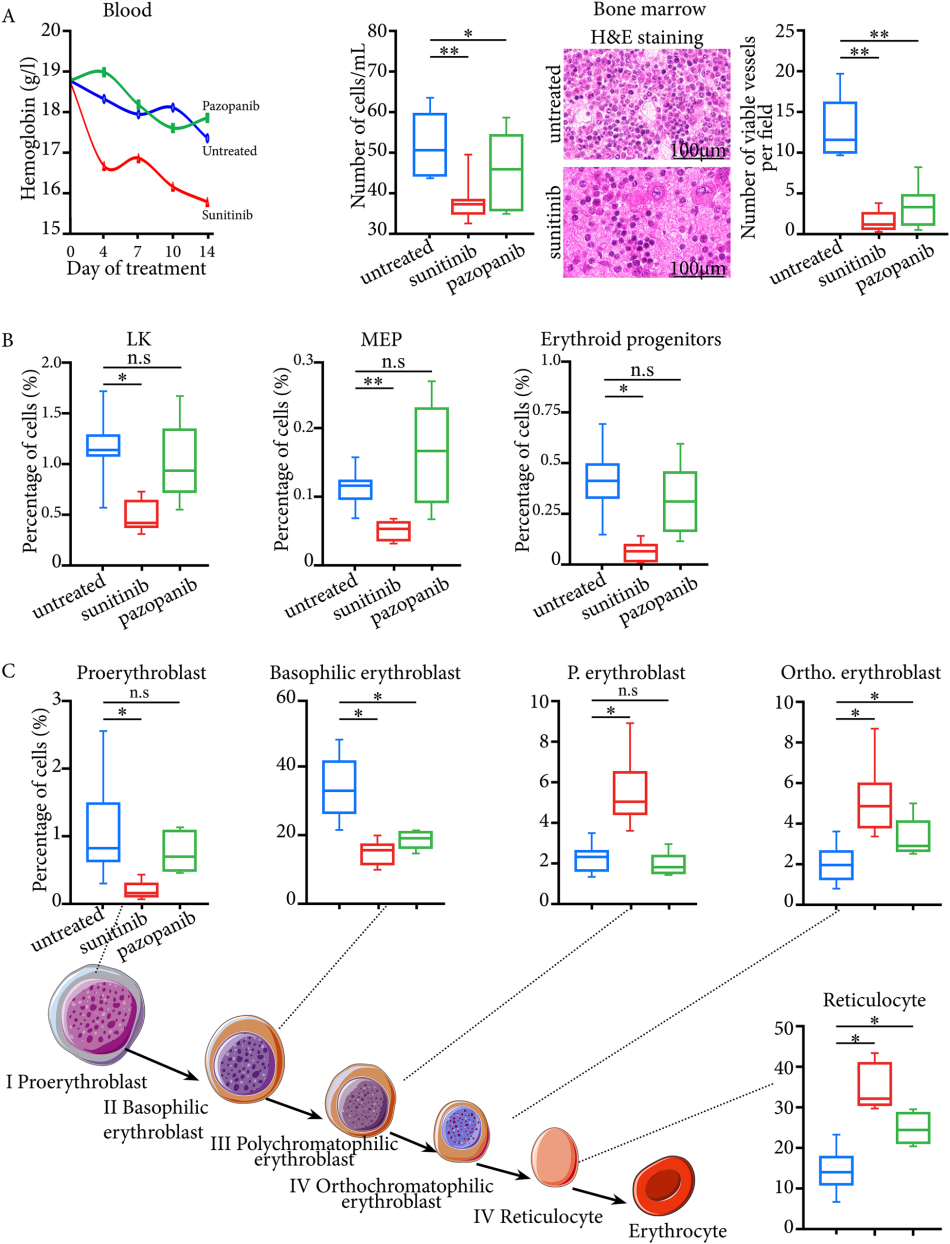
**A** Toxicities induced by anti-angiogenic TKIs are linked to a direct cytotoxic effect on bone-marrow endothelial cells: The left panel shows complete blood count with hemoglobin level and bone-marrow density. The right panel shows H&E staining of bone-marrow sections with a decrease in numbers of microvessels and large haemorrhagic areas under sunitinib treatment. H&E: Hematoxilin-eosin. *: *P* < 0.05; **: *P* < 0.01; n.s: not significant. RQ: Relative quantification. **B** Characterization of the different hematopoietic lineages under anti-angiogenic TKIs using flow cytometry on bone-marrow. It shows a decrease in living cells for the mature LK progenitors, MEP, and erythrocyte progenitors under sunitinib treatment. MEP: Megakaryocyte–erythroid progenitor. **C** Flow cytometry analysis of bone marrow for the different stages of erythrocyte maturation shows a significant decrease in numbers of proerythroblasts and of basophilic erythroblasts under sunitinib treatment, but not for the last three stages, from polychromatophilic erythroblasts to reticulocytes. *: *P* < 0.05; n.s: not significant

Anti-angiogenic tyrosine kinase inhibitors (TKIs) have become major drugs for the treatment of various cancer types [1]. Seven anti-angiogenic TKIs are currently approved, and their toxicities are often limiting their use. In our recent meta-analysis involving 56,895 patients, we reported the high incidence of 56.1% severe toxicities (grades 3–4), pazopanib being the safest drug [2]. Haematological toxicities were particularly frequent using sunitinib (Supp.Fig. 1), and we aimed to decipher the pathophysiology of these toxicities (see Supplementary methods and Supplementary Word 1).

**Fig. 2:**
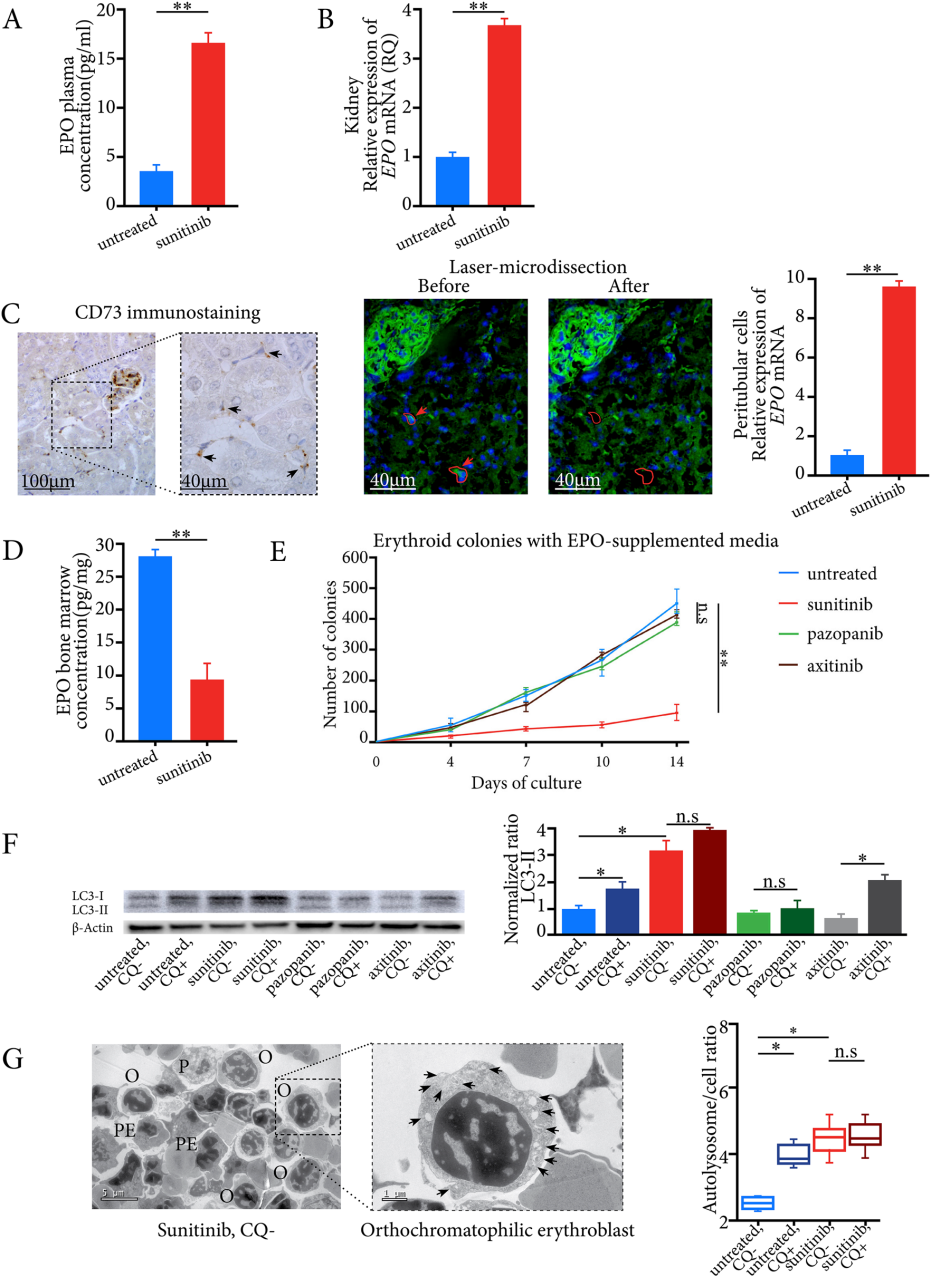
Sunitinib-induced anemia was associated with an adaptative response to systemic hypoxia and autophagy flux inhibition in erythroid progenitors. **A** EPO plasma concentrations in sunitinib-treated mice and untreated mice. **B** mRNA expression levels of *Epo* in the kidneys of sunitinib-treated mice and untreated mice. **C** The left panel shows CD73-expressing peritubular cells on a kidney section from a mouse treated 14 days with sunitinib (black arrows). The right panel illustrates the laser-microdissection of two CD73-expressing renal peritubular cells (green fluorescence, red arrows and continuous lines) on a kidney section from one sunitinib-treated mouse. The right panel shows mRNA quantification of *Epo* in laser-microdissected renal peritubular cells using digital-droplet PCR. **D** Bone marrow EPO concentrations in sunitinib-treated and untreated mice. **E** Cultures of bone-marrow cells from treated and untreated mice using EPO-supplemented media. The numbers of colony-forming units (CFUs) were identified and counted using an inverted phase-contrast microscope at × 40 magnification at Days 0, 4, 7, 10, and 14 of culture. **F** The left panel illustrates immunoblotting for Lc3 with normalised Lc3-II expression in erythroid colonies. β-Actin is shown as a loading control. **G** Transmission electron microscopy images of different erythroid progenitors. The left upper panel illustrates an orthochromatophilic erythroblast at high magnification with numerous autolysosomes (black arrows) from erythroid colonies of sunitinib-treated mice. The left lower panel shows a ratio of autolysosomes to cells, that was significantly higher in erythroid colonies of mice treated with sunitinib compared to untreated mice. P: Proerythroblast; B: Basophilic erythroblast; PE: polychromatophilic erythroblast; O: orthochromatophilic erythroblast. CQ: hydrocloroquine; *: *P* < 0.05; **: *P* < 0.01; n.s: not significant. EPO: erythropoietin; RQ: Relative quantification; RCC: renal cell carcinoma

To model them in mice, we therefore used sunitinib and compared it to pazopanib. After 14 days of treatment, we showed a broad range of toxic effects on normal tissues through a cytotoxic effect on normal endothelial cells including the thyroid, myocardium, and bone-marrow, more marked with sunitinib than pazopanib (Supp.Fig. 2 and Fig. 1A). This was particularly true for bone-marrow, and one striking observation was the complete disappearance of microvessels, with large haemorrhagic areas after 14 days of sunitinib at 40 mg/kg/day (Fig. 1A). Insofar as the effect on bone-marrow was mainly anti-angiogenic, we should have observed a proportional destruction of the different hematopoietic lineages. This was not the case. Using flow cytometry, some cell types were little affected (Supp.Fig. 3, 4). Cell death was particularly marked for the LK and MEP mature progenitors, and then the erythrocyte lineage (Fig. 1B). For EryP progenitors, there was a significant decrease in living cells in sunitinib-treated compared to untreated mice (Fig. 1B). When we focused on the different stages of erythrocyte maturation, we observed a marked decrease in numbers of precursors I and II, whereas there was no cell death in the last three stages, from polychromatophilic erythroblasts to reticulocytes. Proportionally, their numbers increased significantly in sunitinib-treated mice compared to untreated mice (Fig. 1C), suggesting their resistance to hypoxia and a maturation blockage. We then counted circulating reticulocytes in blood and found a significant increase in the immature reticulocyte fraction in sunitinib-treated mice compared to untreated mice at day 14 (0.72 *vs.* 0.44, *P* < 0.05) (Supp. Fig. 5), reinforcing our hypothesis of blockage of erythroid lineage maturation.

We wondered whether this blockage was linked to a poorly adapted response to sunitinib-induced anemia. We found a significant increase in erythropoietin (EPO) plasma levels and in *Epo* mRNA expression levels in whole kidneys of sunitinib-treated mice compared to untreated mice (Fig. 2A,B), confirming an adaptative response to systemic hypoxia. EPO is mainly secreted by renal erythropoietin-producing cells (REPC) in the kidney [3]. Using digital droplet PCR, we showed an approximately ten-fold increase in *Epo* mRNA expression levels of laser-microdissected CD73-expressing REPCs in sunitinib-treated mice compared with untreated mice (Fig. 2C). In contrast, EPO concentrations in total bone-marrow were very low (Fig. 2D), in accordance with the destruction of bone-marrow microvessels, preventing circulating EPO from reaching the bone-marrow at relevant concentrations.

Since erythrocyte maturation is linked to EPO [4], we cultured bone-marrow obtained after 14 days of treatment with sunitinib, using a specific medium containing EPO. We found a significant decrease in numbers of colonies in sunitinib-treated mice compared to untreated mice (Fig. 2E), associated with an accumulation of autofluorescent sunitinib in erythroid colonies (Supp.Fig. 6). EPO was thus no longer sufficient to enable normal erythrocyte maturation and differentiation.

We hypothesized that this EPO-independent maturation blockage could be linked to autophagy flux inhibition secondary to lysosomal sunitinib sequestration [5, 6], the autophagy process being required for erythroid maturation from the stage of polychromatophilic erythroblast [7]. We found a significant increase in *Lc3*, *Bnip3l* and* Becn1* mRNA expression levels as three standard markers of different autophagy stages in erythroid colonies from sunitinib-treated mice (Supp.Fig. 7). In addition, for colonies from untreated mice, Lc3 level significantly increased after exposure to the lysosomotropic agent hydrocloroquine. In erythroid colonies from sunitinib-treated mice, both total Lc3 and Lc3-II protein levels were high and did not increase significantly after chloroquine exposure, suggesting autophagy flux inhibition (Fig. 2F). Using transmission electron microscopy to characterize the different stages of erythrocyte maturation [8], we confirmed the cytometry data and showed that the ratio of autolysosomes/cells was significantly higher in erythroid colonies of sunitinib-treated mice than in those of untreated mice (Fig. 2G). Since sunitinib is the only known lysosomotropic anti-angiogenic TKI, we conducted the same experiments with two other drugs, pazopanib and axitinib. For mice treated with pazopanib and axitinib for 14 days, we first cultured the bone marrows in EPO-containing medium, and evidenced a normal growth of erythroid colonies as in untreated mice (Fig. 2E). In addition, in these colonies, Lc3 protein levels were similar to those observed for untreated mice, which is in favor of an absence of autophagy induction and maturation blockage with these two drugs (Fig. 2F).

We report here the first study to model the overall toxic effects of anti-angiogenic TKIs in mice, while other preclinical studies have mainly focused on cardiac or other specific toxicities [9, 10]. We evidenced that they were due to a diffuse toxic endothelial effect on normal vessels. This is coherent with the physiological expression of VEGFR2 in normal endothelial cells in various tissues, the main target of anti-angiogenic TKIs [11].

Our finding is of particular translational value, since recombinant human EPOs (rhEPOs) are currently approved for the treatment of anemia in cancer [12]. The overexpression of EPO and/or EPO receptor was reported in renal cancer [13] with a possible deleterious effect of rhEPOs on tumor growth and survival [14]. Indeed, in a preclinical murine model of renal cell carcinoma, treatment with rhEPO was associated with tumor progression [15]. Finally, given this potential deleterious effect of rhEPO on tumor growth and its decreased physiological effect on erythrocyte maturation due to sunitinib-induced autophagy flux inhibition (Supp.Fig. 8), we believe that blood transfusion should be considered early on for the treatment of sunitinib-induced anemia.

## Supplementary Information

Supplementary material 1.

Supplementary material 2.

Supplementary material 3.

Supplementary material 4.

Supplementary material 5.

Supplementary material 6.

Supplementary material 7.

Supplementary material 8.

Supplementary material 9.

Supplementary material 10.

## Data Availability

No datasets were generated or analysed during the current study.

## Abbreviations

TKI: Tyrosine kinase inhibitor
EPO: Erythropoietin
VEGFR: Vascular endothelial growth factor receptor
PDGFRβ: Platelet-derived growth factor receptor β
NIH: National Institutes of Health
qRT-PCR: Real-Time Quantitative Reverse Transcription
ddPCR: Droplet Digital Polymerase Chain Reaction
