# Supplementary material for: Anti-angiogenic tyrosine kinase inhibitors and the pathophysiology of their toxic effects: revisiting the treatment of anemia in metastatic cancers
Source: Exp Hematol Oncol. 2025 Apr 19;14:59. doi: 10.1186/s40164-025-00640-9 (PMC12008949; doi:10.1186/s40164-025-00640-9)
Supplement: Supplementary file 1 — Supplementary material 1. [file 40164_2025_640_MOESM1_ESM.docx]

**Anti-angiogenic tyrosine kinase inhibitors and the pathophysiology of their toxic effects: revisiting the treatment of anemia in metastatic cancers**

Tai Van Nguyen^1,2,3,4^, Eurydice Angeli^1,4,5^, Diaddin Hamdan^1,6^, Morad El Bouchtaoui^1,4^, Oanh T. Bui^1,2^, Feriel Azibani^1^, Rong Shen^7^, He Lu^1^, Kien Hung Do^2^, Anne Janin^1^, Quang Van Le^2,3^, Guilhem Bousquet^1,4,5#^

^1^Université de Paris, INSERM, MASCOT, F-75006 Paris, France.

^2^Vietnam National Cancer Hospital – K Hospital, Hanoi, Vietnam.

^3^Hanoi Medical University, Hanoi, Vietnam.

^4^Université Sorbonne Paris Nord, 9 Avenue Jean Baptiste Clément, 93439 Villetaneuse, France.

^5^Assistance Publique Hôpitaux de Paris, Hôpital Avicenne, Service d’oncologie médicale, 93008 Bobigny, France.

^6^Hôpital La Porte Verte, Versailles F-78004, France.

^7^Department of Hematology, Shanghai Institute of Hematology, Ruijin Hospital Affiliated to Shanghai Jiao Tong University School of Medicine. No. 197, Ruijin 2nd Road, Shanghai, 200025.

**Corresponding author:** Prof. Guilhem Bousquet, UMR_S942 Inserm-Université de Paris-Université Paris 13, UFR SMBH, 1 rue Chablis 93000 Bobigny; e-mail: [guilhem.bousquet@aphp.fr](mailto:guilhem.bousquet@aphp.fr)

**Funding**

This study was supported by the Bourse d’Excellence de l’Ambassade de France au Viet Nam (grant number: 133125S).

**Abstract**

**Background**: Anti-angiogenic tyrosine kinase inhibitors (TKIs) have become major drugs for the treatment of various cancer types, but with an overall high incidence of severe toxicities, particularly haematological toxicities including severe anemia.

**Methods**: We treated C57BL6 mice continuously by gavage for 14 days with either sunitinib, pazopanib, or axitinib. In this study, we set out to decipher the pathophysiological mechanisms of anti-angiogenic TKI haematological toxicity.

**Results**: We demonstrated that anti-angiogenic TKIs induced a broad range of toxic effects on normal tissues through a cytotoxic effect on normal endothelial cells. Haematological toxicities were particulary marked with sunitinib. Sunitinib-induced hypoxia through the destruction of normal vessels in the bone marrow mainly affected erythrocyte and myeloid lineages, and this was associated with a blockage in erythrocyte maturation. Althought sunitinib-induced anemia was associated with an adaptative response to systemic hypoxia, we demonstrated that erythropoietin (EPO) concentrations in the total bone marrow of sunitinib-treated mice were significantly lower than in untreated mice. This is coherent with the destruction of microvessels in the bone marrow under sunitinib treatment, preventing circulating EPO from reaching the bone marrow at relevant concentrations. However, we demonstrated an additional effect specific to sunitinib that induced autophagy flux inhibition in erythroid progenitors, with a blockage of erythrocyte maturation, leading to more severe anemia.

**Conclusions**: We deciphered the pathophysiology of anti-angiogenic TKI-induced anemia, which we observed to be mainly linked to a direct effect on normal bone-marrow vessels and to autophagy flux inhibition in erythroid progenitors under sunitinib.

**Keywords**: anti-angiogenic agents, tyrosine kinase inhibitor, anemia, sunitinib, autophagy, erythropoietin.

**Introduction**

In the last ten years, anti-angiogenic tyrosine kinase inhibitors (TKIs) have become major drugs for the treatment of various cancer types, mainly renal cancer, hepatocarcinoma, thyroid and colorectal cancers [1–3]. Seven anti-angiogenic TKIs are currently approved, and their toxicities are well described. These toxicities are often severe and limit their use. In particular, they often lead to a decrease in doses, and therefore to the possible development of resistance linked to insufficient pharmacological exposure. In a recent meta-analysis involving 56,895 patients with any cancer type, we reported the generally high incidence of 56.1% severe toxicities (grades 3-4), pazopanib being the safest drug. The most frequent toxicities are hypertension, hand-foot syndrome, fatigue, and haematological toxicities [4]. Fatigue, occuring in up to 60% of patients treated with anti-angiogenic TKIs, considerably alters their quality of life [5], and is partly explained by anemia which for its part occurs in up to 50% of patients, in particular with sunitinib [6]. The pathophysiological mechanisms of haematological toxicities have not been deciphered to date.

All anti-angiogenic TKIs have an anti-tumor effect mainly by way of an anti-angiogenic effect on tumor neovessels, although a direct effect on tumor cells has also been reported [7–9]. In a previous preclinical study, we showed that the pro-apoptotic effect of sunitinib on tumor endothelial cells was linked to the expression of the theoretical targets of sunitinib, including VEGFR1/2 and PDGFRβ [9]. Preclinical studies using anti-angiogenic TKIs have shown a direct effect on normal microvessels in the small intestin and pancreas [10–12]. Since VEGFRs/PDGFRs are ubiquitously expressed in normal endothelial cells [13, 14], we hypothesized that anti-angiogenic TKIs could have a diffuse cytotoxic effect on normal vessels in various organs, including the bone marrow.

In this study, we set out to understand the pathophysiological mechanisms of anti-angiogenic TKI haematological toxicity.

**Material and Methods**

**Data extracted from a meta-analysis**

We extracted data from our recent meta-analysis on anti-angiogenic TKI toxicities, focusing on haematological toxicities [4]. Our meta-analysis was registered in PROSPERO under number CRD42023411946.

**Mouse models**

C57BL6 mice, purchased from Janvier (France), were maintained in pathogen-free animal housing in the Sorbonne Paris Nord University (agreement number C9300801). All experiments were perfomed in accordance with NIH guidelines, and the European Union recommendations (2010/63/UE). This study was approved by the National French Ethics Committee for experimental animal studies (APAFIS#17190-201810181424511).

To model in mice the toxicities of anti-angiogenic TKIs observed in patients, we treated mice continuously by gavage for 14 days either with sunitinib at a pharmacologically relevant dose of 40mg/kg/day [15], or with pazopanib at a dose of 150mg/kg/day diluted in 0.9% NaCl [16, 17] or with axitinib at a dose of 25mg/kg/day [18]. Untreated C57BL6 mice were used as the control group.

**Complete blood count monitoring**

Peripheral blood was obtained before treatment at Day 0, at three time-points during treatment (Day 4, Day 7, Day 10), and at the time of euthanasia (Day 14). The blood was immediately placed in ethylenediaminetetraacetic acid (EDTA)-coated tubes, and hemograms were performed using the MS9-5 analyser.

**Manual reticulocyte counts**

Manual counts of reticulocytes were performed after automated monitoring of complete blood counts. 30 μl of cresyl blue (Sigma-Aldrich®, Germany) and 30 μl of blood sample were gently mixed in a test tube and were incubated at room temperature for 20 minutes. After remixing, a thin smear was prepared. One thousand red cells were counted on each smear of all samples. The percentage of cells containing stained RNA was recorded using an Olympus® BX51 microscope (Japan). Cells containing abundant RNA were identified as immature reticulocytes or early reticulocytes, while the remaining reticulocytes were identified as mature reticulocytes or normal reticulocytes. The immature reticulocyte fraction (IRF) was calculated as the ratio of immature reticulocytes to the total number of reticulocytes, enabling assessment of erythroid lineage maturation.

**Assessement of cardiac toxicity**

For each mouse, echocardiography parameters were measured using transthoracic echocardiography at Day 13, the day before euthanasia, on the ACUSON S3000 TM (Siemens) Cardiovascular Ultrasound system equipped with a 14-MHz linear transducer. The mice were mildly sedated with ketamine (0.125 mg/kg) during measurements. All echocardiography investigations were performed blind and verified by a second operator.

At the time of euthanasia (Day 14), the hearts were immediately dissected and divided into two parts for tissue analyses: i) one was formaldehyde-fixed and paraffin-embedded, ii) one was snap-frozen in liquid nitrogen.

*Cardiac Bnp* mRNA expression was assessed using qRT-PCR as described below.

**Tissue analyses and immunostaining**

At euthanasia, all organs were systematically dissected for further tissue analyses. All tissue samples were similarly processed and divided into two parts: one was formaldehyde-fixed and paraffin-embedded, one was snap-frozen in liquid nitrogen. All tissue sections were obtained using an HM-340 automatic microtome (Microm) set at 2 µm, and they were stained with Hematoxylin-Eosin (HE). Two pathologists (A.J., G.B.) performed the tissue analyses.

For thyroid analysis, colloid surface areas were delineated and measured using CellSens Dimension software (Olympus, Germany) on five different fields at ×200 magnification using a Provis AX70 microscope (Olympus, Tokyo). For each thyroid, the total colloid surface area was expressed as a percentage of the total thyroid surface area.

For the hearts and thyroids, microvessel density was assessed on tissue sections using CD31 monoclonal rat anti-mouse antibody (Dianova, Germany, clone SZ31) at 1:30 dilution as the primary antibody. An indirect immunoperoxidase method was used, and controls had no primary antibody or an irrelevant primary antibody of the same isotype. The secondary antibody was a rabbit anti-rat IgG H&L (ab6703,1:200, Abcam), coupled with an anti-rabbit OmniMap detection kit (Roche diagnostic, Meylan, France). The number of stained blood vessels was counted on five different fields at ×400 magnification, on a Provis-aX-70 microscope (Olympus, Tokyo). The results were expressed as mean ± standard error of the mean (SeM).

For bone-marrow microvessel density, normal vessels without disruption of the basal membrane were counted on five different fields at ×400 magnification, on a Provis-aX-70 microscope (Olympus, Tokyo).

Renal erythropoietin-producing cells were identified using CD73 polyclonal rabbit anti-mouse antibody at 1:100 dilution (abcam, UK, ab175396), as the primary antibody. An indirect immunoperoxidase method was used, and controls had no primary antibody or an irrelevant primary antibody of the same isotype.

**Flow cytometry**

For each mouse, at the time of euthanasia (Day 14), bone marrow was aspirated from femurs and suspended in PBS/SVF 2%. Red Blood Cell Lysis Buffer (Sigma Aldrich) was used before staining. Hematopoietic stem cells (HSC), multipotent hematopoietic progenitors (MPP), myeloid progenitors (MP), granulocyte monocyte precursors (PreGM), granulocyte-monocyte progenitors (GMP), megakaryocyte erythroid progenitors (MEP), erythroid progenitors (EryP), and megakaryocyte progenitors (MkP) were stained with a panel of rat anti-mouse antibodies (all Sony Biotechnologies) including the PacBlue lineage cocktail (B220, Ter-119, CD3e, Gr-1, CD11b), PercpCy5.5 CD117 (Clone 2B8), PE/Cy7 Sca-1(Clone D7), BUV737 CD16/32 (Clone 2.4G2), Brilliant Violet 650 CD150 (Clone TC15-12F12.2), Alexa Fluor 647 CD105 (Clone MJ7/18), and Brilliant Violet 605 CD41 (Clone MWReg30). All experiments were performed using a FACs LSR Fortessa (BD Biosciences) and analysed with FlowJo® software (FlowJo, LLC, USA).

For each type of progenitor, results were expressed as a percentage of the total number of living cells in the bone-marrow analysed.

**Assessement of plasma and bone-marrow EPO concentrations**

For EPO concentration in plasma, blood was centrifuged for 10 min at 1000×g within 30 min of collection. EPO concentrations were assessed on 50 μL of plasma, using a mouse erythropoietin ELISA kit (BioLegend, Canada, 442707).

For EPO concentrations in the bone marrow, bone marrow was lysed in 200 μL of RIPA buffer (Thermofisher) and the EPO concentration was determined using the same ELISA kit.

**mRNA quantification using qRT-PCR and ddPCR**

For *Bnp* and *Epo* mRNA expression in tissues, total RNA was extracted from 10 frozen 10 μm sections, using RNeasy Mini Kit (Qiagen, France), it was quantified on NanoDrop (LabTech, France) and qualified on Bio-Rad ExperionTM Automated Electrophoresis Station, France. Total RNA was reverse-transcribed (cDNA) before qPCR amplification using random primers SuperScriptTM II Reverse Transcriptase (Invitrogen, France). The qPCR reactions were run using fluorescent probes on a GeneAmpPCR System (Applied Biosystems) with specific primers *Bnp* (Mm01255770_g1, Thermofisher) or *Epo* (Mm01202755_m1, Thermofisher). The housekeeping genes *Tbp* (Mm01277042_m1, Thermofisher) and *Gapdh* (Mm99999915_g1, Thermofisher) were used to normalize gene expression results. The results were expressed as -∆CT and 2-∆∆CT (also known as relative quantification, RQ).

For *Epo* mRNA expression assessment in renal erythropoietin-producing cells (REPC), 7 µm-thick frozen renal tissue sections were fixed in 70% ethanol for 20 sec, and then labeled with anti CD73 (ab175396, abcam, UK) APEX™ Antibody Labeling Alexa Fluo 488 Kit (A10468, Thermofisher, Germany) at 1/20 concentration for 2 minutes at 4°C. For each sample, one hundred peritubular CD73-expressing cells were immediately laser-microdissected using a PALM-Microbeam/Zeiss system (Carl Zeiss, Germany), and processed for RNA extraction and reverse-transcription (cDNA) before Droplet Digital Polymerase Chain Reaction (ddPCR). DdPCR was performed using the QX100 ddPCR workflow system (Biorad, Hercules, CA, USA). The mix contained 5 µL cDNA from RT-PCR, 10µL of ddPCR Supermix for Probes (No dUTP) (Bio Rad), 1µL of *Epo* probes (Mm01202755_m1, Themofisher) per well, and the final volume for the reaction was 20μL. Droplets were generated by a QX200 Droplet Generator (Biorad). PCR was carried out on the CFX96 Real Time System (Bio Rad). PCR was performed with an initial denaturing step at 95°C for 10mn, followed by 40 cycles of denaturing (94°C for 30s), and annealing (60°C for 1mn). A post-amplification melting curve program was initiated by heating to 98°C for 10mn and then cooling down to 12°C. Each PCR run included a no-template control. The results of ddPCR were generated using QX100 Droplet Reader (Biorad) and analysed using QuantaSoft software (Biorad).

**Erythroid colony formation assay**

For each mouse, whether or not treated with TKIs, the two humerus bones free of skin and muscle tissues were cracked and ground. Bone marrow cells were inoculated in a 35mm tissue culture plate at 2x10^5^ cells per 3 ml of methylcellulose-based medium (MethoCult™ GF M3334), purchased from STEMCELL Technologies Inc. (Canada). The cells were incubated at 37°C in the presence of 5% CO_2_. The numbers of colony-forming units (CFUs) were identified and counted using an inverted phase-contrast microscope at ×40 magnification at Days 0, 4, 7, 10, and 14 of culture. Duplicate plates were also prepared.

**Assessment of autophagy markers**

After 10 days of culture, erythroid colonies were processed for qRT-PCR and Western Blot. For autophagy flux inhibition, erythroid colonies were incubated with hydrocloroquine at 10 µmol/L for 2h before collection for further analysis [19].

qRT-PCR was perfomed as described above to assess mRNA expression of *Lc3* (Mm00458725_g1, Thermofisher), *Becn1* (Mm01265461_m1, Thermofisher), and *Bnip3l* (Mm00786306_s1, Thermofisher).

Protein extraction was performed using RIPA buffer (Thermo) supplemented with 1 EDTA-free protease inhibitor cocktail tablet (Roche Diagnostics) and phosphatase inhibitors (Sigma-Aldrich). Western blot was performed on 4-20% Mini-PROTEAN TGX precast gels (Biorad), then transferred to 0.2 µm PVDF membrane (Biorad) using the Trans-Blot Turbo Transfer System. Immunostaining was performed using anti-LC3 (PA5-22990, 1/1000, Thermofisher) and anti-β Actin (A3854, 1/50,000, Sigma) as primary antibodies. An anti-rabbit HRP (ab32568, 1/1000, abcam) was used as the second antibody. Development was performed using Clarity Western ECL Substrate (Biorad) and read on ChemiDoc XRS+ detection system (Biorad). Analyses were performed using Image Lab Software 6.1 (Biorad).

**Transmission electron microscopy (TEM)**

Erythroid colonies were processed for transmission electron microscopy after 10 days of culture. Erythroid colonies were centrifuged at 1000 rpm for 10 minutes and fixed with 2% glutaraldehyd in 0.1 M of sodium cacodylate buffer. After ethanol dehydration and Epon-embedding of fixed samples, semi-thin sections were prepared. Ultrathin sections (150nm) were obtained on selected zones, stained and analyzed on a Hitachi H-7650 TEM microscope at 80 kV.

**Statistical analysis**

The data was analyzed using R software (version 4.1.0, R Foundation for Statistical Computing, Vienna, Austria; http://www.r-project.org).

Quantitative variables were expressed as mean ± standard deviation (SD) and categorical variables as numbers and percentages. By reference for untreated group (controls), a two-by-two comparison with each TKI was performed using the Student’s t test. P values under 0.05 were considered significant.

**Results**

**Sunitinib presents the poorest profile in terms of haematological toxicities for patients**

Using unpublished data from our recent meta-analysis on anti-angiogenic TKI toxicities [4], we focused on haematological toxicities (all grades and grades 3-4 in particular) in 86 trials involving 16,877 patients. When we considered all grades, the prevalence of haematological toxicities ranged from 9.4% (neutropenia under regorafenib treatment) to 47.9% (leucopenia under sunitinib treatment). When we considered severe grades 3-4 toxicities alone, sunitinib had the highest implication for all four biological parameters, in particular for anemia, where it reached 5.2% (Supp.Fig1).

To model anti-angiogenic TKI toxicities in preclinical murine models, we therefore chose sunitinib. We decided to compare its effects with those of pazopanib, which presents the safest profile for patients among all seven anti-angiogenic TKIs.

**Sunitinib and pazopanib induce diffuse endothelial toxicity in normal vessels**

We treated ten C57BL6 mice continuously with sunitinib for 14 days at a pharmacologically relevant dose of 40mg/kg/day [15]. Ten untreated C57BL6 mice were used as the control group. Apart from haematological toxicites, we focused on heart and thyroid analyses since cardiovascular (hypertension, heart failure) and endocrine toxicities (hypothyroidism, asthenia) are frequent and possibly severe [4]. For cardiac toxicity, all mice had transthoracic echocardiography the day before euthanasia. At the time of euthanasia, all organs were systematically analysed. When we compared sunitinib-treated mice with untreated mice, we observed a significant decrease in the percentage of colloid thyroid surface (Supp.Fig2A). Using ultrasonogrpahy, we also found a significant decrease in the left ventricular ejection fraction (LVEF) (*P* < 0.01), and a signifciant increase in cardiac mRNA expression of *Bnp* (*P* < 0.01), a biomarker of cardiac dysfunction (Supp.Fig2B). For haematological toxicity, sunitinib-treated mice developed anemia, with a significant decrease in hemoglobin and leucocyte counts compared to untreated mice, whereas platelet counts increased significantly (*P* < 0.05) (Fig1A). Of note, a decrease in hematoglobin level was also observed for untreated mice, due to repeated blood sampling over 14 days. However, under sunitinib treatment, anemia was even more severe. We then analysed bone marrow and observed a significant decrease in bone marrow density in treated mice compared to untreated mice, with a total median number of cells of 16x10^6^ *vs.* 35x10^6^ cells respectively (*P* < 0.01) (Fig1A).

When we assessed microvessel density in all these tissues, we constantly observed a significant decrease in numbers of CD31-expressing normal vessels in sunitinib-treated mice compared to untreated mice (*P* < 0.01) (Supp.Fig2, right panels). This was particularly true for bone-marrow, and one striking observation was the complete disappearance of microvessels, with large haemorrhagic areas after 14 days of sunitinib at 40mg/kg/day (Fig1A, right panel).

We also treated ten mice with pazopanib and likewise observed a significant decrease in normal vessel counts in the thyroid, the heart and the bone-marrow of treated mice, although these effects were less marked than with sunitinib. In particular, in the bone-marrow of mice treated with pazopanib, the microvessels were still present. However, when we counted only normal vessels without disruption of the basal membrane, their number was significantly smaller than in the untreated mice (3±2 per field at magnification x400 *vs*. 12±8, *P* < 0.01) (Fig1A, right panel).

Overall, anti-angiogenic TKIs induce a broad range of toxic effects on normal tissues through a cytotoxic effect on normal endothelial cells.

**The effect of sunitinib on the different blood cell lineages is not proportional.**

Insofar as the effect on bone-marrow of sunitinib or pazopanib was mainly anti-angiogenic, we should have observed a proportional bone-marrow destruction of the different hematopoietic lineages using flow cytometry. This was not the case. Some cell types were little or not at all affected, including the most immature LSK progenitors (hematopoietic stem cells (HSC) and multipotent progenitors (MPP)), and also platelets, granulocytes, and lymphocytes (Supp.Fig3). This could be linked to an intrinsic resistance to hypoxic stress of normal hematopoietic stem-cells [20–22], and also of lymphocytes and platelets [23, 24]. In contrast, cell death was particularly marked for the LK mature progenitors, including erythrocyte and myeloid lineages. For MEP progenitors, we observed a 45% decrease (0.05%±0.013% in living cells for sunitinib-treated mice *vs.* 0.11%±0.02% for untreated mice, *P* < 0.001). For EryP, there was a 80% decrease (0.09%±0.07% in living cells *vs.* 0.48%±0.1% for sunitinib treated *vs.* untreated; *P* < 0.001) (Fig1B). In contrast, MKP progenitors, which also derive from MEP progenitors, were not significantly affected by sunitinib treatment (Supp.Fig4).

Overall, sunitinib-induced hypoxia following the destruction of normal vessels in bone marrow mainly affects erythrocyte and myeloid lineages.

**The effect of sunitinib is associated with a blockage of erythrocyte maturation.**

When we focused on the different stages of erythrocyte maturation, we observed a marked decrease in numbers of proerythroblasts and of basophilic erythroblasts (precursors I and II) in the bone marrow, whereas there was no cell death in the last three stages, from polychromatophilic erythroblasts to reticulocytes. Proportionally, their numbers increased significantly in mice treated with sunitinib compared to untreated mice (Fig1C), suggesting their resistance to hypoxia and a blockage of maturation. Again, these effects were less marked with pazopanib. In particular, the number of polychromatophilic erythroblasts (precursors III) did not increase compared to the numbers among untreated mice.

We then counted circulating reticulocytes in blood, both immature and mature, using Cresyl blue specific stain, and showed a significant increase in their total absolute count. We found a significant increase in the immature reticulocyte fraction (IRF) in sunitinib-treated mice compared to untreated mice at day 14 (0.72 *vs.* 0.44, *P* < 0.05), reinforcing our hypothesis of blockage of erythroid lineage maturation. No significant difference was observed for IRF among pazopanib-treated mice compared to untreated mice (Supp.Fig5).

**Sunitinib-induced anemia was associated with an adaptative response to systemic hypoxia**

All further experiments were performed using only sunitinib treatment.

We wondered whether this blockage of erythrocyte maturation was linked to a poorly adapted response to sunitinib-induced anemia and systemic hypoxia. When we measured erythropoietin (EPO) plasma levels, we found a significant increase among sunitinib-treated mice compared to untreated mice (16.4 *vs.* 3.9 pg/ml, *P* < 0.01) (Fig2A). This was associated with a significant increase in *Epo* mRNA expression levels in whole kidneys of mice treated with sunitinib compared to untreated mice, confirming an adaptative response to systemic hypoxia (Fig2B).

Since EPO is mainly secreted by renal erythropoietin-producing cells (REPC) in the kidney [25–28], we assessed *Epo* mRNA expression in these specific cells. Using laser microdissection to select CD73-expressing REPCs combined with digital droplet PCR, we compared sunitinib-treated mice with untreated mice. We showed an approximately ten-fold increase in *Epo* mRNA expression levels (Fig2C).

We finally measured EPO concentrations in total bone marrow of sunitinib-treated mice and found a significant decrease compared to untreated mice (9.4±4.6 *vs.* 26.7±4.3 pg/mg, *P* < 0.01) (Fig2D). This is coherent with the destruction of bone marrow microvessels under sunitinib treatment, preventing circulating EPO from reaching the bone marrow at relevant concentrations.

**Multifactorial blockage of erythrocyte maturation**

Since erythrocyte maturation in the bone marrow is linked to EPO [29, 30], we cultured bone marrow obtained after 14 days of treatment with sunitinib, using a specific medium containing EPO. When we counted erythroid colonies after 10 days of culture, we found a significant decrease in their numbers in sunitinib-treated mice compared to untreated mice (92.6±12.6 *vs.* 440.7±23.5 colonies/dish, *P* < 0.01) (Fig2E). This result suggests that, at this stage, EPO was no longer sufficient to enable normal erythrocyte maturation and differentiation.

Since sunitinib is known to be autofluorescent, we showed that it accumulated in erythroid colonies in sunitinib-treated mice. No autofluorescent was observed in untreated mice (Supp.Fig6).

In the light of previous preclinical studies on renal cancer cells [31, 32] and normal endothelial cells [33], we hypothesized that this EPO-independent maturation blockage could be linked to autophagy flux inhibition secondary to lysosomal sunitinib sequestration. Indeed, the autophagy process is required for erythroid maturation into erythrocytes from the stage of polychromatophilic erythroblast [34], enabling chromatin and mitochondria expulsion from terminal erythroid progenitors. As recommended, we studied autophagy using complementary methods. We first assessed mRNA expression level of three standard markers of different autophagy stages. We found a significant increase in *Lc3*, *Bnip3l* and *Becn1* mRNA expression levels in erythroid colonies from mice treated with sunitinib compared to untreated mice (Supp.Fig7A). To demonstrate that it was associated with autophagy flux inhibition, we measured total Lc3 protein (Lc3-I and Lc3-II) in erythroid colonies with or without exposure to hydrocloroquine (a lysosomotropic agent) using Western blot [19]. As expected for colonies from untreated mice, Lc3 level significantly increased after chloroquine exposure. We also found that both total Lc3 and Lc3-II protein levels were higher in erythroid colonies from mice treated with sunitinib than in those of untreated mice. However, the level did not increase significantly after chloroquine exposure, suggesting autophagy flux inhibition (Fig2F, normalized data only shown for Lc3-II).

Using transmission electron microscopy to characterize the different stages of erythrocyte maturation [19, 35], we found that there was a larger proportion of mature erythroblasts (polychromatophilic and orthochromatophilic) in mice treated with sunitinib compared to untreated mice (Supp.Fig7B). In addition, after treatment of erythroid colonies with chroloquine for one hour on day 10, the distribution of erythroid progenitors was similar to that observed with sunitinib alone. When we assessed the ratio of autolysosomes/cells, it was significantly higher in erythroid colonies of mice treated with sunitinib than in those of untreated mice (Fig2G and Supp.Fig7C).

Since sunitinib is the only known lysosomotropic anti-angiogenic TKI, we conducted the same experiments with two other drugs, pazopanib and axitinib. For mice treated with pazopanib and axitinib for 14 days, we first cultured the bone marrows in EPO-containing medium, and evidenced a normal growth of erythroid colonies as in untreated mice (Fig2E). In addition, in these colonies, Lc3 protein levels were similar to those observed for untreated mice, which is in favor of an absence of autophagy induction and maturation blockage with these two drugs (Fig2F).

To sum up, sunitinib-induced autophagy flux inhibition in erythroid progenitors led to a blockage in erythrocyte maturation, leading to more severe anemia.

**Discussion**

We report here the first study to model the overall toxic effects of anti-angiogenic TKIs in mice, while other preclinical studies have mainly focused on cardiac or other specific toxicities [10–12]. We have evidenced that these effects were due to a diffuse toxic endothelial effect on normal vessels. This is coherent with the physiological expression of VEGFR2 in normal endothelial cells in various tissues, the main target of anti-angiogenic TKIs [36–38]. We had previously shown, in cancers, that the effect of sunitinib was linked to the expression of VEGFR1/2, PDGFRα/β by cancer cells [9].

Here we intentionally focused on haematological toxicity, since it is frequent and limits the use of anti-angiogenic TKIs in daily clinical practice [39, 40]. Indeed, in view of hitherto unpublished data from our recent meta-analysis on anti-angiogenic TKI toxicities among 56,985 patients with cancers, the prevalence of all-grade haematological toxicities was high, reaching almost 50% [4]. We also evidenced considerable differences across the seven approved drugs, among which sunitinib is the most haematotoxic and pazopanib the safest drug. We set out to determine the pathophysiological reasons for these differences. Using a preclinical murine model, we showed that haematological toxicity, particularly anemia, was linked to a direct effect on normal bone marrow vessels, but this was not the only finding. In particular, there was a striking difference between sunitinib and pazopanib. Indeed, for sunitinib, we showed that anemia was associated with a blockage of erythrocyte maturation despite an adaptative renal response to systemic hypoxia. We demonstrated that this maturation blockage was linked to autophagy flux inhibition in erythroid progenitors in sunitinib-treated mice, and that this effect was specifically linked to sunitinib and not to the other anti-angiogenic TKIs tested (pazopanib, axitinib). This is a major result, coherent with the fact that treatment with sunitinib is associated with the highest prevalence of all-grade anemia, and also with previously described effects of sunitinib on cancer cells and normal endothelial cells, linked to the lysosomal sequestration of sunitinib [31–33]. This is also coherent with our observation of a blokage of maturation from the stage of polychromatophilic erythroblasts, when the physiological autophagy process starts for erythroid terminal maturation [34]. As electronic microscopy is the gold standard for autophagy assessment [19], we counted the total numbers of cytoplasmic vesicules, as recommended. This enabled us to confirm the existence of autophagy flux inhibition with sunitinib treatment, with erythroid maturation blockage from the stage of polychromatophilic erythroblasts. Autophagy is crucial in maintaining balance in mammalian cells by recycling cytoplasmic components. This process involves the formation of autophagosomes, which capture cell organelles and cytoplasmic materials, and then merge with lysosomes to break down the contents of the autophagosomes. Physiologically, autophagy plays a critical role in the elimination of mitochondria, ribosomes and other organelles during erythroid terminal differentiation [41–43] and the authophagic flux inhibiton during erythropoiesis led to a blockage of erythrocyte maturation, and thus anemia [43, 44]. In our study, we thus demonstrated that sunitinib-induced severe anemia was directly linked to autophagy flux inhibition in erythroid progenitors.

Our finding is of particular translational value, since recombinant human EPOs (rhEPOs) are currently approved for the treatment of anemia in cancer [45–49]. However, no study has focused specfically on metastatic renal cell carcinoma. In addition, various clinical studies have reported the overexpression of EPO and/or EPO receptor in renal cancer [50–52] with a possible deleterious effect of rhEPOs on tumor growth and survival [52–55]. Indeed, in a preclinical murine model of renal cell carcinoma, treatment with rhEPO was associated with tumor progression [56].

Finally, given this potential deleterious effect of rhEPO on tumor growth and its decreased physiological effect on erythrocyte maturation due to sunitinib-induced autophagy flux inhibition, we believe that blood transfusion should be considered early on for the treatment of sunitinib-induced anemia, so as to decrease possible deleterious effects on tumor growth linked to systemic hypoxia and secondary physiological EPO production.

In our study, we deciphered the pathophysiology of anti-angiogenic TKI-induced anemia, which we found to be mainly linked to a direct effect on bone-marrow normal vessels and also to autophagy flux inhibition in erythroid progenitors under sunitinib (Supp.Fig8). Our study has potential translational applications for the choice of anti-angiogenic TKIs and the management of treatment-induced anemia.

**Abbreviations**

TKI, tyrosine kinase inhibitor; EPO, erythropoietin; VEGFR, Vascular endothelial growth factor receptor ; PDGFRβ, Platelet-derived growth factor receptor β; NIH, National Institutes of Health, ; qRT-PCR, Real-Time Quantitative Reverse Transcription; ddPCR, Droplet Digital Polymerase Chain Reaction; Bnp, B-type Natriuretic Peptide.

**Declarations**

**Ethics approval and consent to participate**

This study was approved by the National French Ethics Committee for experimental animal studies (APAFIS#17190-201810181424511).

**Consent for publication**

NA

**Availability of data and materials**

No datasets were generated or analysed during the current study.

**Competing interests**

The authors have no competing interests to report.

**Funding**

This study was supported by the Bourse d’Excellence de l’Ambassade de France au Viet Nam (grant number: 133125S).

**Authors' contributions**

Conception and design: G. B.

Development of the methodology: T.V.N. and G. B.

Acquisition of data: All authors.

Analyses and interpretation of data: T.V.N and G. B.

Drafting and review: T.V.N. and G. B.

Revision of the manuscript: All authors.

Material support: T.V.N, M.B., O.T.B, F.A., H.L., and G.B.

Administrative and technical: G. B.

Study supervision: G. B.

**Acknowledgements**

We thank Ms. Angela Swaine for the revision of the English language.

**References**

1. Manz KM, Fenchel K, Eilers A, et al (2020) Efficacy and Safety of Approved First-Line Tyrosine Kinase Inhibitor Treatments in Metastatic Renal Cell Carcinoma: A Network Meta-Analysis. Adv Ther 37:730–744. https://doi.org/10.1007/s12325-019-01167-2

2. Llovet JM, Ricci S, Mazzaferro V, et al (2008) Sorafenib in advanced hepatocellular carcinoma. N Engl J Med 359:378–390. https://doi.org/10.1056/NEJMoa0708857

3. Grothey A, Van Cutsem E, Sobrero A, et al (2013) Regorafenib monotherapy for previously treated metastatic colorectal cancer (CORRECT): an international, multicentre, randomised, placebo-controlled, phase 3 trial. Lancet Lond Engl 381:303–312. https://doi.org/10.1016/S0140-6736(12)61900-X

4. Van Nguyen T, Hamdan D, Falgarone G, et al (2024) Anti-Angiogenic Tyrosine Kinase Inhibitor-Related Toxicities Among Cancer Patients: A Systematic Review and Meta-Analysis. Target Oncol. https://doi.org/10.1007/s11523-024-01067-8

5. Escudier B, Porta C, Bono P, et al (2014) Randomized, controlled, double-blind, cross-over trial assessing treatment preference for pazopanib versus sunitinib in patients with metastatic renal cell carcinoma: PISCES Study. J Clin Oncol Off J Am Soc Clin Oncol 32:1412–1418. https://doi.org/10.1200/JCO.2013.50.8267

6. Funakoshi T, Latif A, Galsky MD (2013) Risk of hematologic toxicities in cancer patients treated with sunitinib: a systematic review and meta-analysis. Cancer Treat Rev 39:818–830. https://doi.org/10.1016/j.ctrv.2013.01.004

7. Gotink KJ, Verheul HMW (2010) Anti-angiogenic tyrosine kinase inhibitors: what is their mechanism of action? Angiogenesis 13:1–14. https://doi.org/10.1007/s10456-009-9160-6

8. Al-Abd AM, Alamoudi AJ, Abdel-Naim AB, et al (2017) Anti-angiogenic agents for the treatment of solid tumors: Potential pathways, therapy and current strategies - A review. J Adv Res 8:591–605. https://doi.org/10.1016/j.jare.2017.06.006

9. Bousquet G, Varna M, Ferreira I, et al (2013) Differential regulation of sunitinib targets predicts its tumor-type-specific effect on endothelial and/or tumor cell apoptosis. Cancer Chemother Pharmacol 72:1183–1193. https://doi.org/10.1007/s00280-013-2300-0

10. Kamba T, McDonald DM (2007) Mechanisms of adverse effects of anti-VEGF therapy for cancer. Br J Cancer 96:1788–1795. https://doi.org/10.1038/sj.bjc.6603813

11. Baffert F, Le T, Sennino B, et al (2006) Cellular changes in normal blood capillaries undergoing regression after inhibition of VEGF signaling. Am J Physiol Heart Circ Physiol 290:H547-559. https://doi.org/10.1152/ajpheart.00616.2005

12. Kamba T, Tam BYY, Hashizume H, et al (2006) VEGF-dependent plasticity of fenestrated capillaries in the normal adult microvasculature. Am J Physiol Heart Circ Physiol 290:H560-576. https://doi.org/10.1152/ajpheart.00133.2005

13. Raica M, Cimpean AM (2010) Platelet-Derived Growth Factor (PDGF)/PDGF Receptors (PDGFR) Axis as Target for Antitumor and Antiangiogenic Therapy. Pharmaceuticals 3:572–599. https://doi.org/10.3390/ph3030572

14. Shibuya M (2011) Vascular Endothelial Growth Factor (VEGF) and Its Receptor (VEGFR) Signaling in Angiogenesis: A Crucial Target for Anti- and Pro-Angiogenic Therapies. Genes Cancer 2:1097–1105. https://doi.org/10.1177/1947601911423031

15. Mendel DB, Laird AD, Xin X, et al (2003) In vivo antitumor activity of SU11248, a novel tyrosine kinase inhibitor targeting vascular endothelial growth factor and platelet-derived growth factor receptors: determination of a pharmacokinetic/pharmacodynamic relationship. Clin Cancer Res Off J Am Assoc Cancer Res 9:327–337

16. Hashimoto K, Man S, Xu P, et al (2010) Potent Preclinical Impact of Metronomic Low-Dose Oral Topotecan Combined with the Antiangiogenic Drug Pazopanib for the Treatment of Ovarian Cancer. Mol Cancer Ther 9:996. https://doi.org/10.1158/1535-7163.MCT-09-0960

17. Reguera-Nuñez E, Man S, Xu P, Kerbel RS (2018) Preclinical impact of high dose intermittent antiangiogenic tyrosine kinase inhibitor pazopanib in intrinsically resistant tumor models. Angiogenesis 21:793–804. https://doi.org/10.1007/s10456-018-9623-8

18. Fenton BM, Paoni SF (2007) The Addition of AG-013736 to Fractionated Radiation Improves Tumor Response without Functionally Normalizing the Tumor Vasculature. Cancer Res 67:9921–9928. https://doi.org/10.1158/0008-5472.CAN-07-1066

19. Klionsky DJ, Abdel-Aziz AK, Abdelfatah S, et al (2021) Guidelines for the use and interpretation of assays for monitoring autophagy (4th edition)(1). Autophagy 17:1–382. https://doi.org/10.1080/15548627.2020.1797280

20. Kocabas F, Xie L, Xie J, et al (2015) Hypoxic metabolism in human hematopoietic stem cells. Cell Biosci 5:39. https://doi.org/10.1186/s13578-015-0020-3

21. Eliasson P, Jönsson J-I (2010) The hematopoietic stem cell niche: Low in oxygen but a nice place to be. J Cell Physiol 222:17–22. https://doi.org/10.1002/jcp.21908

22. Koller MR, Bender JG, Miller WM, Papoutsakis ET (1992) Reduced oxygen tension increases hematopoiesis in long-term culture of human stem and progenitor cells from cord blood and bone marrow. Exp Hematol 20:264–270

23. Chabi S, Uzan B, Naguibneva I, et al (2019) Hypoxia Regulates Lymphoid Development of Human Hematopoietic Progenitors. Cell Rep 29:2307-2320.e6. https://doi.org/10.1016/j.celrep.2019.10.050

24. Paterson GG, Young JM, Willson JA, et al (2020) Hypoxia Modulates Platelet Purinergic Signalling Pathways. Thromb Haemost 120:253–261. https://doi.org/10.1055/s-0039-3400305

25. Eckardt KU, Ratcliffe PJ, Tan CC, et al (1992) Age-dependent expression of the erythropoietin gene in rat liver and kidneys. J Clin Invest 89:753–760. https://doi.org/10.1172/JCI115652

26. Yamazaki S, Souma T, Hirano I, et al (2013) A mouse model of adult-onset anaemia due to erythropoietin deficiency. Nat Commun 4:1950. https://doi.org/10.1038/ncomms2950

27. Pan X, Suzuki N, Hirano I, et al (2011) Isolation and characterization of renal erythropoietin-producing cells from genetically produced anemia mice. PloS One 6:e25839. https://doi.org/10.1371/journal.pone.0025839

28. Obara N, Suzuki N, Kim K, et al (2008) Repression via the GATA box is essential for tissue-specific erythropoietin gene expression. Blood 111:5223–5232. https://doi.org/10.1182/blood-2007-10-115857

29. Zivot A, Lipton JM, Narla A, Blanc L (2018) Erythropoiesis: insights into pathophysiology and treatments in 2017. Mol Med Camb Mass 24:11–11. https://doi.org/10.1186/s10020-018-0011-z

30. Malik J, Kim AR, Tyre KA, et al (2013) Erythropoietin critically regulates the terminal maturation of murine and human primitive erythroblasts. Haematologica 98:1778–1787. https://doi.org/10.3324/haematol.2013.087361

31. Giuliano S, Cormerais Y, Dufies M, et al (2015) Resistance to sunitinib in renal clear cell carcinoma results from sequestration in lysosomes and inhibition of the autophagic flux. Autophagy 11:1891–1904. https://doi.org/10.1080/15548627.2015.1085742

32. Gotink KJ, Broxterman HJ, Labots M, et al (2011) Lysosomal sequestration of sunitinib: a novel mechanism of drug resistance. Clin Cancer Res Off J Am Assoc Cancer Res 17:7337–7346. https://doi.org/10.1158/1078-0432.CCR-11-1667

33. Wu S, Huang L, Shen R, et al (2020) Drug resistance‑related sunitinib sequestration in autophagolysosomes of endothelial cells. Int J Oncol 56:113–122. https://doi.org/10.3892/ijo.2019.4924

34. Betin VMS, Singleton BK, Parsons SF, et al (2013) Autophagy facilitates organelle clearance during differentiation of human erythroblasts: evidence for a role for ATG4 paralogs during autophagosome maturation. Autophagy 9:881–893. https://doi.org/10.4161/auto.24172

35. Dussouchaud A, Jacob J, Secq C, et al (2022) Transmission Electron Microscopy to Follow Ultrastructural Modifications of Erythroblasts Upon ex vivo Human Erythropoiesis. Front Physiol 12:

36. Feng D, Nagy JA, Brekken RA, et al (2000) Ultrastructural localization of the vascular permeability factor/vascular endothelial growth factor (VPF/VEGF) receptor-2 (FLK-1, KDR) in normal mouse kidney and in the hyperpermeable vessels induced by VPF/VEGF-expressing tumors and adenoviral vectors. J Histochem Cytochem Off J Histochem Soc 48:545–556. https://doi.org/10.1177/002215540004800412

37. Witmer AN, Dai J, Weich HA, et al (2002) Expression of vascular endothelial growth factor receptors 1, 2, and 3 in quiescent endothelia. J Histochem Cytochem Off J Histochem Soc 50:767–777. https://doi.org/10.1177/002215540205000603

38. Simon M, Röckl W, Hornig C, et al (1998) Receptors of vascular endothelial growth factor/vascular permeability factor (VEGF/VPF) in fetal and adult human kidney: localization and [125I]VEGF binding sites. J Am Soc Nephrol JASN 9:1032–1044. https://doi.org/10.1681/ASN.V961032

39. Funakoshi T, Latif A, Galsky MD (2013) Risk of hematologic toxicities in cancer patients treated with sunitinib: a systematic review and meta-analysis. Cancer Treat Rev 39:818–830. https://doi.org/10.1016/j.ctrv.2013.01.004

40. Saiyed M, Shah C (2015) Hematological Toxicities Associated with Pazopanib use in Cancer Patients: A Meta-Analysis. Value Health 18:A816. https://doi.org/10.1016/j.jval.2015.09.231

41. Sandoval H, Thiagarajan P, Dasgupta SK, et al (2008) Essential role for Nix in autophagic maturation of erythroid cells. Nature 454:232–235

42. Chen M, Sandoval H, Wang J (2008) Selective mitochondrial autophagy during erythroid maturation. Autophagy 4:926–928

43. Zhang J, Wu K, Xiao X, et al (2015) Autophagy as a Regulatory Component of Erythropoiesis. Int J Mol Sci 16:4083–4094. https://doi.org/10.3390/ijms16024083

44. Lupo F, Tibaldi E, Matte A, et al (2016) A new molecular link between defective autophagy and erythroid abnormalities in chorea-acanthocytosis. Blood 128:2976–2987. https://doi.org/10.1182/blood-2016-07-727321

45. Rodríguez Sánchez CA (2007) Recommendation of the scientific societies on the treatment of anaemia in cancer patients. Clin Transl Oncol Off Publ Fed Span Oncol Soc Natl Cancer Inst Mex 9:582–589. https://doi.org/10.1007/s12094-007-0107-3

46. Aapro M, Beguin Y, Bokemeyer C, et al (2018) Management of anaemia and iron deficiency in patients with cancer: ESMO Clinical Practice Guidelines†. Ann Oncol 29:iv96–iv110. https://doi.org/10.1093/annonc/mdx758

47. Musio F (2020) Revisiting the treatment of anemia in the setting of chronic kidney disease, hematologic malignancies, and cancer: perspectives with opinion and commentary. Expert Rev Hematol 13:1175–1188. https://doi.org/10.1080/17474086.2020.1830371

48. Tonelli M, Hemmelgarn B, Reiman T, et al (2009) Benefits and harms of erythropoiesis-stimulating agents for anemia related to cancer: a meta-analysis. CMAJ Can Med Assoc J J Assoc Medicale Can 180:E62–E71. https://doi.org/10.1503/cmaj.090470

49. Bohlius J, Schmidlin K, Brillant C, et al (2009) Recombinant human erythropoiesis-stimulating agents and mortality in patients with cancer: a meta-analysis of randomised trials. Lancet Lond Engl 373:1532–1542. https://doi.org/10.1016/S0140-6736(09)60502-X

50. Ito K, Yoshii H, Asano T, et al (2012) Impact of increased erythropoietin receptor expression and elevated serum erythropoietin levels on clinicopathological features and prognosis in renal cell carcinoma. Exp Ther Med 3:937–944. https://doi.org/10.3892/etm.2012.513

51. Michael A, Politi E, Havranek E, et al (2007) Prognostic significance of erythropoietin expression in human renal cell carcinoma. BJU Int 100:291–294. https://doi.org/10.1111/j.1464-410X.2007.06978.x

52. Westenfelder C, Baranowski RL (2000) Erythropoietin stimulates proliferation of human renal carcinoma cells. Kidney Int 58:647–657. https://doi.org/10.1046/j.1523-1755.2000.00211.x

53. Ljungberg B, Rasmuson T, Grankvist K (1992) Erythropoietin in renal cell carcinoma: evaluation of its usefulness as a tumor marker. Eur Urol 21:160–163. https://doi.org/10.1159/000474825

54. Henke M, Laszig R, Rübe C, et al (2003) Erythropoietin to treat head and neck cancer patients with anaemia undergoing radiotherapy: randomised, double-blind, placebo-controlled trial. Lancet Lond Engl 362:1255–1260. https://doi.org/10.1016/S0140-6736(03)14567-9

55. Leyland-Jones B (2003) Breast cancer trial with erythropoietin terminated unexpectedly. Lancet Oncol 4:459–460. https://doi.org/10.1016/S1470-2045(03)01163-X

56. Nakamura M, Zhang Y, Yang Y, et al (2017) Off-tumor targets compromise antiangiogenic drug sensitivity by inducing kidney erythropoietin production. Proc Natl Acad Sci U S A 114:E9635–E9644. https://doi.org/10.1073/pnas.1703431114

**Figure legends:**

**Fig1 :** A) Toxicities induced by anti-angiogenic TKIs are linked to a direct cytotoxic effect on bone-marrow endothelial cells: The left panel shows complete blood count with hemoglobin level and bone-marrow density. The right panel shows H&E staining of bone-marrow sections with a decrease in numbers of microvessels and large haemorrhagic areas under sunitinib treatment. H&E: Hematoxilin-eosin. *: *P* < 0.05; **: *P* < 0.01; n.s: not significant. RQ: Relative quantification.

B) Characterization of the different hematopoietic lineages under anti-angiogenic TKIs using flow cytometry on bone-marrow. It shows a decrease in living cells for the mature LK progenitors, MEP, and erythrocyte progenitors under sunitinib treatment. MEP: Megakaryocyte–erythroid progenitor

C) Flow cytometry analysis of bone marrow for the different stages of erythrocyte maturation shows a significant decrease in numbers of proerythroblasts and of basophilic erythroblasts under sunitinib treatment, but not for the last three stages, from polychromatophilic erythroblasts to reticulocytes. *: *P* < 0.05; n.s: not significant.

**Fig2:** Sunitinib-induced anemia was associated with an adaptative response to systemic hypoxia and autophagy flux inhibition in erythroid progenitors.

A) EPO plasma concentrations in sunitinib-treated mice and untreated mice.

B) mRNA expression levels of *Epo* in the kidneys of sunitinib-treated mice and untreated mice.

C) The left panel shows CD73-expressing peritubular cells on a kidney section from a mouse treated 14 days with sunitinib (black arrows). The right panel illustrates the laser-microdissection of two CD73-expressing renal peritubular cells (green fluorescence, red arrows and continuous lines) on a kidney section from one sunitinib-treated mouse. The right panel shows mRNA quantification of *Epo* in laser-microdissected renal peritubular cells using digital-droplet PCR.

D) Bone marrow EPO concentrations in sunitinib-treated and untreated mice.

E) Cultures of bone-marrow cells from treated and untreated mice using EPO-supplemented media. The numbers of colony-forming units (CFUs) were identified and counted using an inverted phase-contrast microscope at ×40 magnification at Days 0, 4, 7, 10, and 14 of culture.

F) The left panel illustrates immunoblotting for Lc3 with normalised Lc3-II expression in erythroid colonies. β-Actin is shown as a loading control.

G) Transmission electron microscopy images of different erythroid progenitors. The left upper panel illustrates an orthochromatophilic erythroblast at high magnification with numerous autolysosomes (black arrows) from erythroid colonies of sunitinib-treated mice. The left lower panel shows a ratio of autolysosomes to cells, that was significantly higher in erythroid colonies of mice treated with sunitinib compared to untreated mice. P: Proerythroblast; B: Basophilic erythroblast; PE: polychromatophilic erythroblast; O: orthochromatophilic erythroblast.

CQ: hydrocloroquine; *: *P* < 0.05; **: *P* < 0.01; n.s: not significant. EPO: erythropoietin; RQ: Relative quantification; RCC: renal cell carcinoma.

**Supplementary Figure legends:**

**Supp.Fig1:** Diagrammatic representation of anti-angiogenic TKIs for the treatment of cancers according to haematological toxicities in a meta-analysis on 16,877 patients: A) all grades and B) grades 3-4 toxicities.

**Supp.Fig2:** Toxicities induced by anti-angiogenic TKIs are linked to a direct cytotoxic effect on normal endothelial cells.

A) The left panel shows H&E staining of thyroid sections with a significant decrease in the percentage of colloid surface area under sunitinib treatment. The right panel shows CD31 immunostaining of thyroid sections with a significant decrease in number of CD31-expressing normal vessels in sunitinib-treated mice compared to untreated mice. B) The left panel shows cardiac toxicity with a significant decrease in the left ventricular ejection fraction visualised on echocardiography, and a signifciant increase in cardiac mRNA expression of *Bnp.* The right panel shows CD31 immunostaining of heart sections with a significant decrease in number of CD31-expressing normal vessels in sunitinib-treated mice compared to untreated mice.

*: *P* < 0.05; **: *P* < 0.01; n.s: not significant. RQ: Relative quantification.

**Supp.Fig3:** Diagrammatic representation of hematopoietic stem cells and the proportion of different hematopoietic lineages using flow cytometry on bone-marrow. No significant difference is observed in living cells for LSK, HSC, MPP, thrombocytes, granulocytes, B cells, and T cells. LSK: Lin^-^Sca1^+^c-Kit^+^; LK: Lin^−^ cKit^+^; LT-HSC: Long-Term Hematopoietic Stem Cell; ST-HSC: Short-Term Hematopoietic Stem Cell; MPP: Multipotential Progenitor; MEP: Megakaryocyte–erythroid progenitor; CLP: Common Lymphoid Progenitor; preGMP: granulocyte-monocyte precursor; GMP: granulocyte-monocyte progenitor; EryP: erythroid progenitor; MKP: megakaryocyte progenitor. n.s: not significant.

**Supp.Fig4:** Characterization of the different hematopoietic lineages under anti-angiogenic TKIs using flow cytometry on bone-marrow.

The left panel provides a diagrammatic representation of the production of mature blood cells from hematopoietic stem cells. The right panel shows the proprotions of different hematopoietic lineages using flow cytometry with a nonsignificant difference in living cells for the PreGMP, MKP and GMP progenitors under sunitinib and pazopanib treatment. LSK: Lin^-^Sca1^+^c-Kit^+^; LK: Lin^−^ cKit^+^; LT-HSC: Long-Term Hematopoietic Stem Cell; ST-HSC: Short-Term Hematopoietic Stem Cell; MPP: Multipotential Progenitor; MEP: Megakaryocyte–erythroid progenitor; CLP: Common Lymphoid Progenitor; preGMP: granulocyte monocyte precursor; GMP: granulocyte-monocyte progenitor; EryP: erythroid progenitor; MKP: megakaryocyte progenitor. *: *P* < 0.05; **: *P* < 0.01; n.s: not significant.

Supp.Fig5: Reticulocyte count shows a significant increase in immature reticulocyte fractions in sunitinib-treated mice compared to untreated mice. *: *P* < 0.05; n.s: not significant.

**Supp.Fig6:** Sunitinib autofluorescence in erythroid colonies from untreated and sunitinib-treated mice (yellow, red arrows).

**Supp.Fig7:** Sunitinib-induced anemia was linked to autophagy flux inhibition in erythroid progenitors.

A) The left panel shows mRNA expression levels of *Bnip3l, Becn1* in erythroid colonies from sunitinib-treated and untreated mice. The right panel shows mRNA expression levels of *Lc3* in erythroid colonies with or without exposure to hydrocloroquine (CQ). B) Counts of erythroid progenitors in erythroid colonies using transmission electron microscopy.

P: Proerythroblast; B: Basophilic erythroblast; PE: polychromatophilic erythroblast; O: orthochromatophilic erythroblast.

CQ: hydrocloroquine; *: *P* < 0.05; **: *P* < 0.01; n.s: not significant.

**Supp.Fig8:** Diagrammatic representation of the pathophysiology of anti-angiogenic TKI-induced anemia. PCE: Polychromatic erythrocyte; ARNT: Aryl Hydrocarbon Receptor Nuclear Translocator, HIF: Hypoxia-inducible Factor; EPO: Erythropoietin; GFR, Vascular endothelial growth factor receptor ; PDGFRβ, Platelet-derived growth factor receptor β; HRE: Hypoxia-Responsive Elements.
