# Supplementary figures and images for: Anti-angiogenic tyrosine kinase inhibitors and the pathophysiology of their toxic effects: revisiting the treatment of anemia in metastatic cancers
Source: Exp Hematol Oncol. 2025 Apr 19;14:59. doi: 10.1186/s40164-025-00640-9 (PMC12008949; doi:10.1186/s40164-025-00640-9)

A

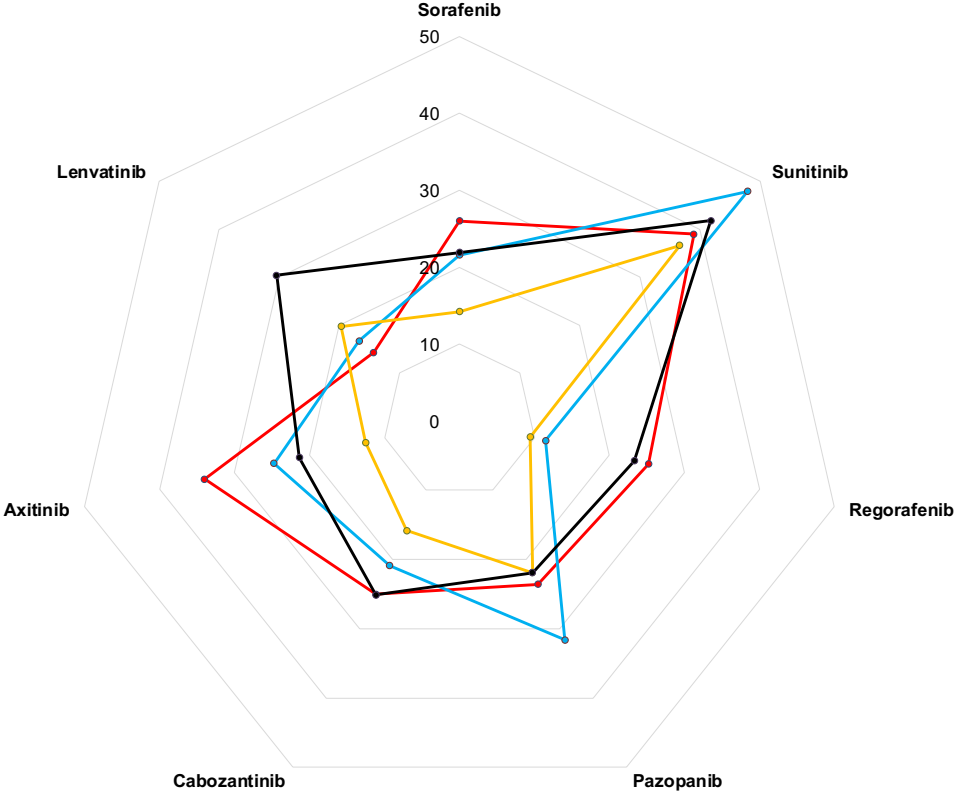

B

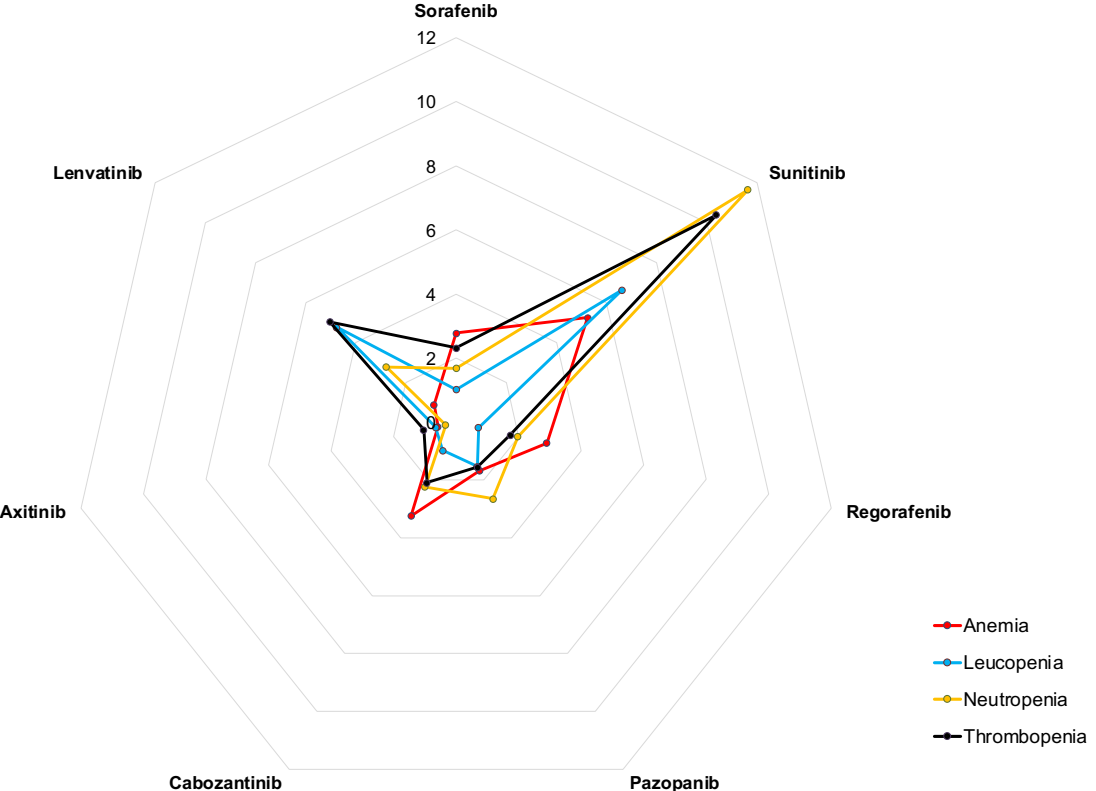

Supplement: Supplementary file 2 — Supplementary material 2. [file 40164_2025_640_MOESM2_ESM.pdf]

## A Thyroid

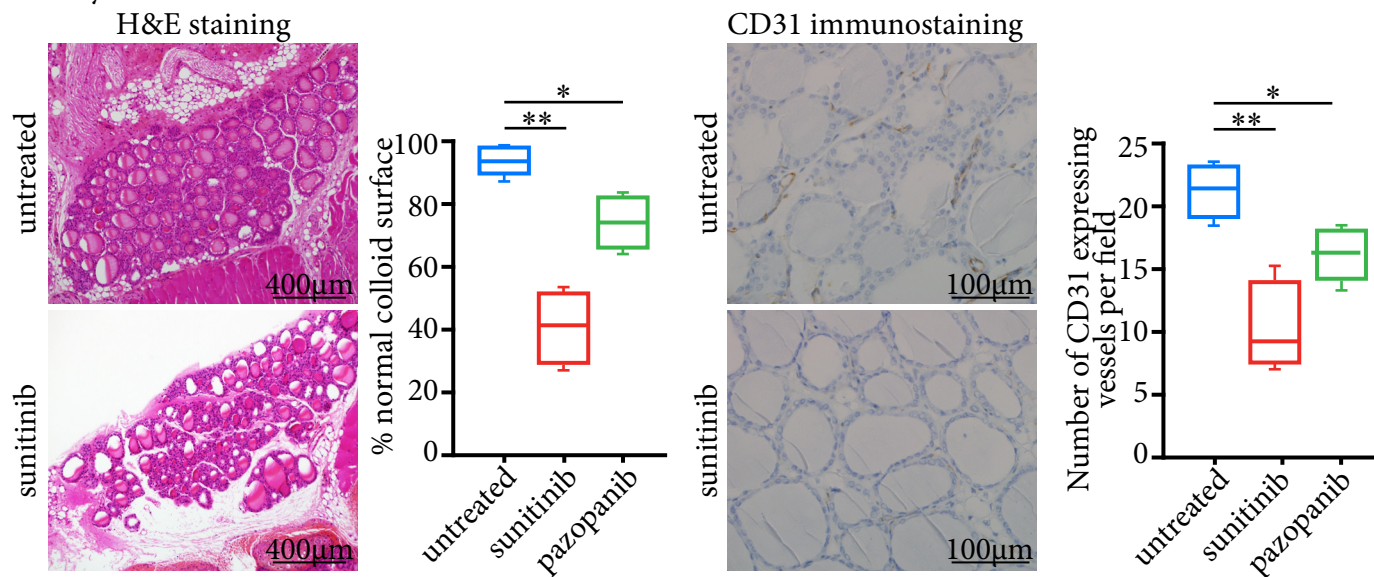

## B Heart

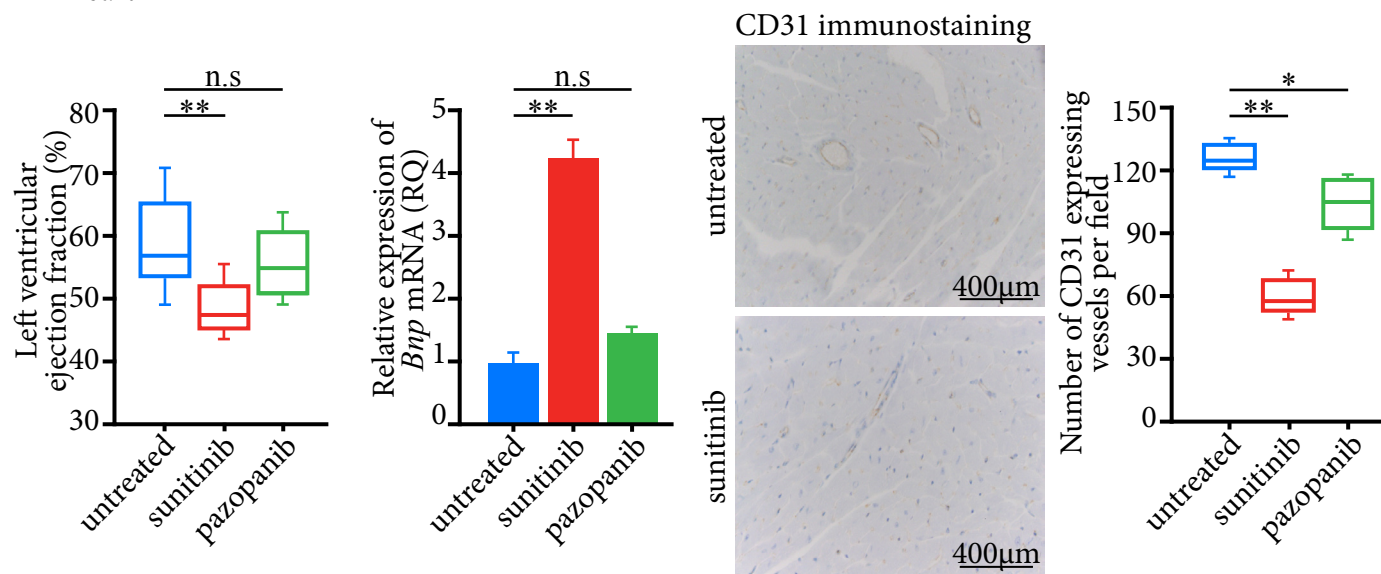

Supplement: Supplementary file 3 — Supplementary material 3. [file 40164_2025_640_MOESM3_ESM.pdf]

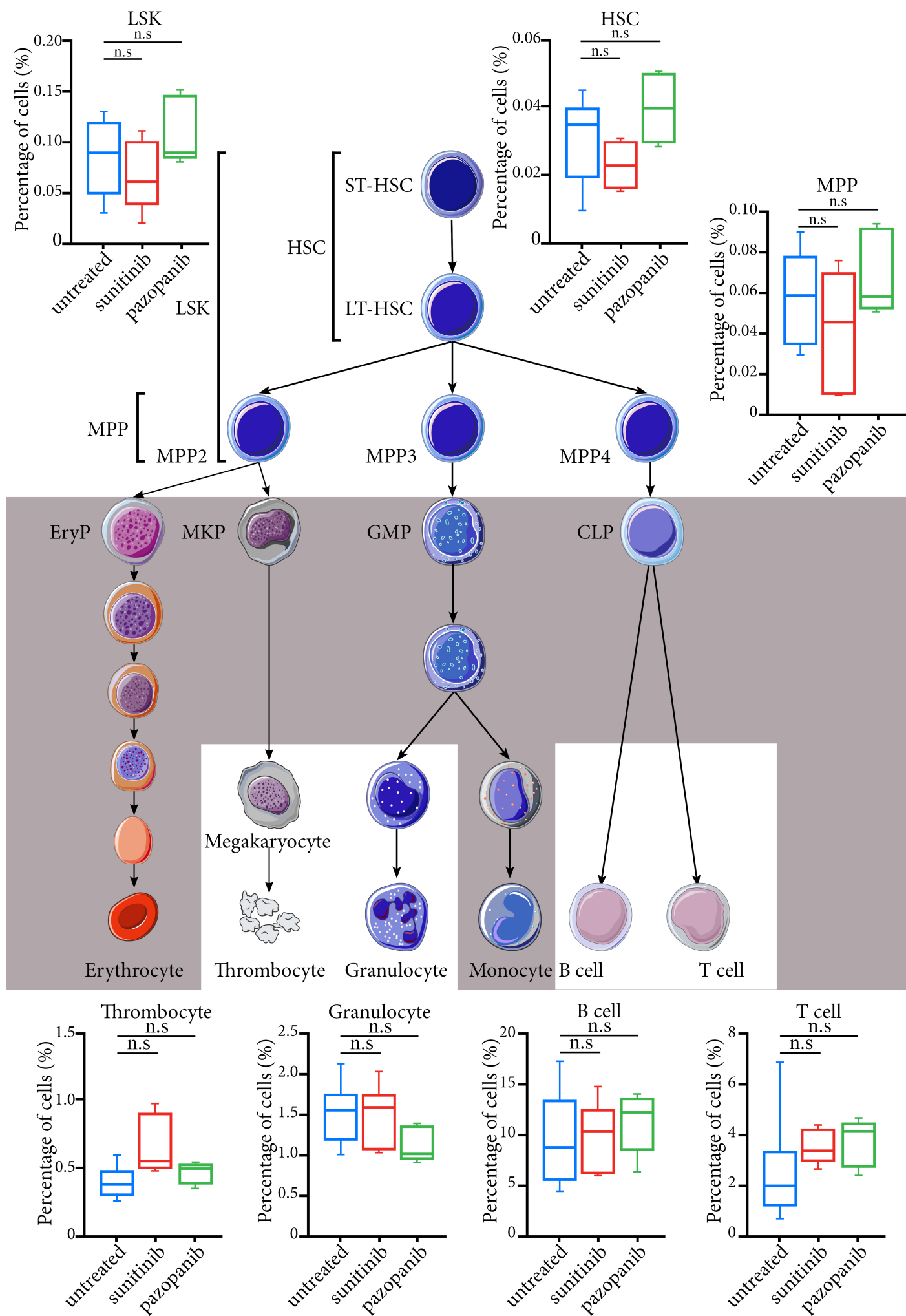

Supplement: Supplementary file 4 — Supplementary material 4. [file 40164_2025_640_MOESM4_ESM.pdf]

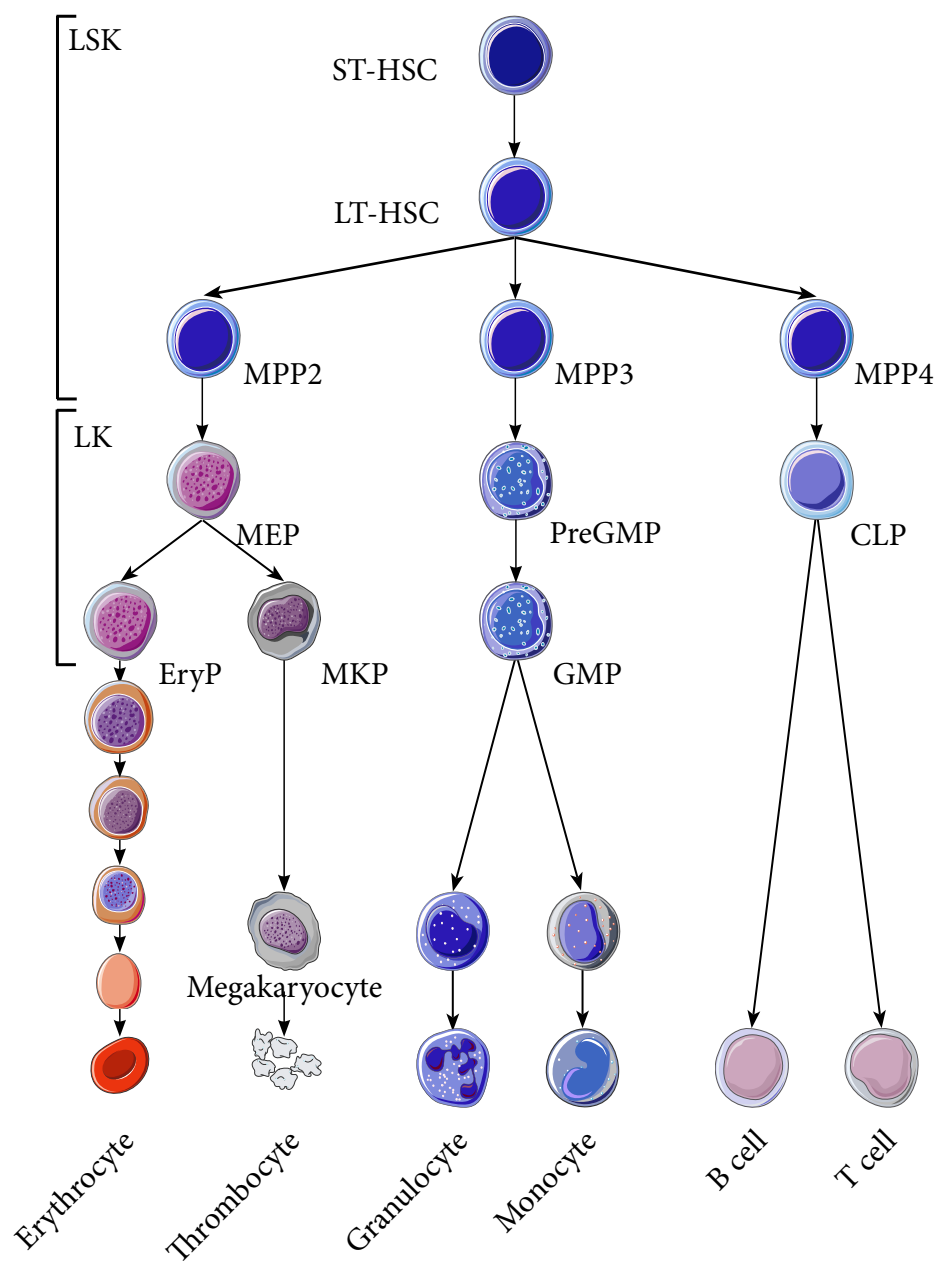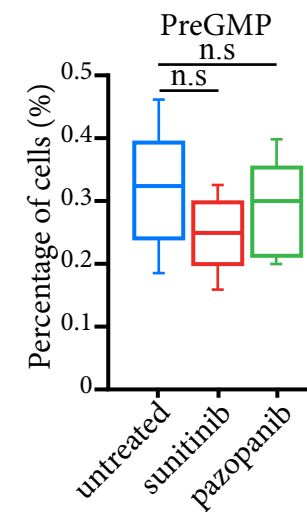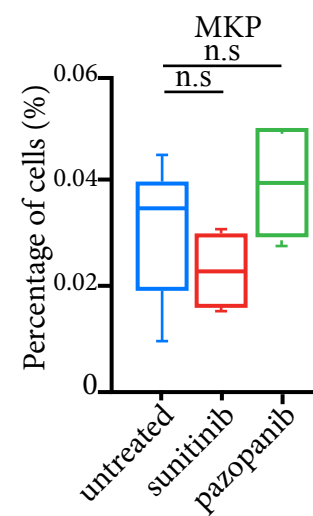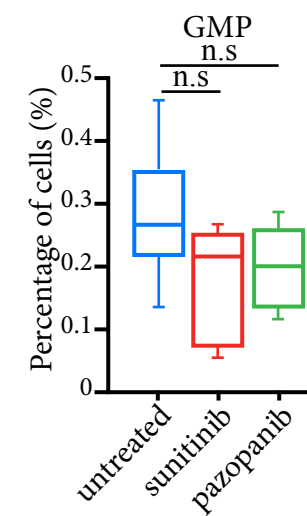

Supplement: Supplementary file 5 — Supplementary material 5. [file 40164_2025_640_MOESM5_ESM.pdf]

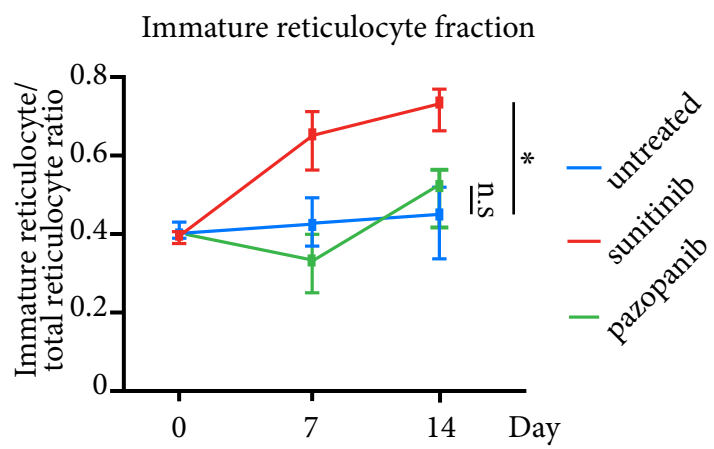

Supplement: Supplementary file 6 — Supplementary material 6. [file 40164_2025_640_MOESM6_ESM.pdf]

## Sunitinib autofluorescence

Erythroblasts from  
erythroid colonies

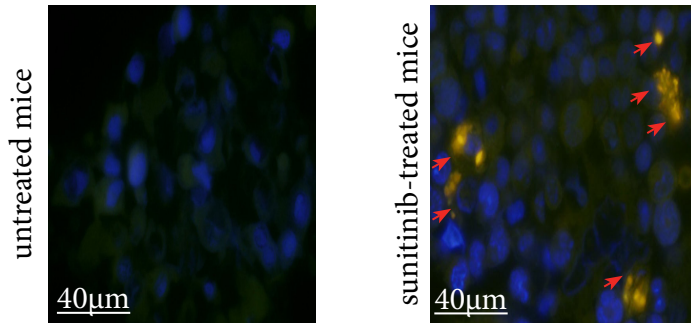

Supplement: Supplementary file 7 — Supplementary material 7. [file 40164_2025_640_MOESM7_ESM.pdf]

A

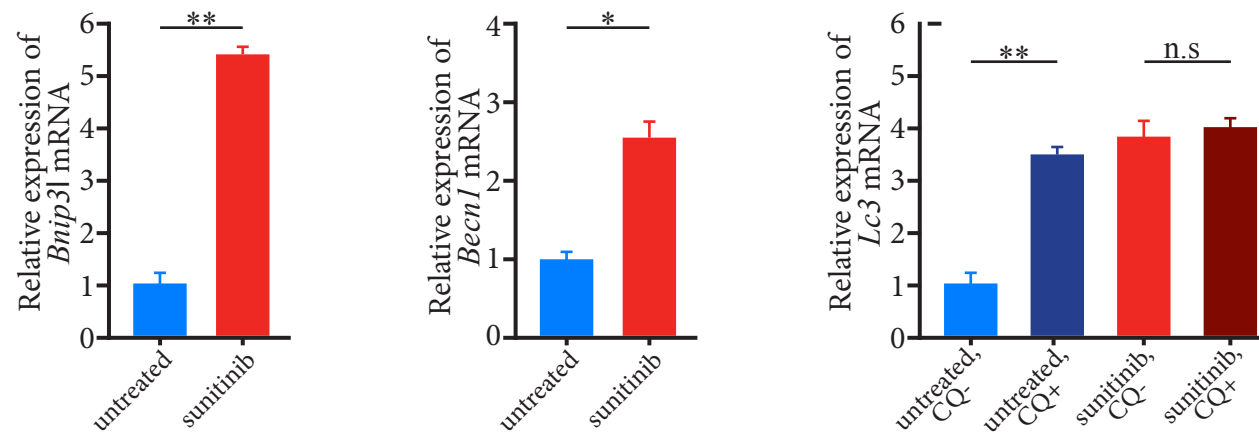

B

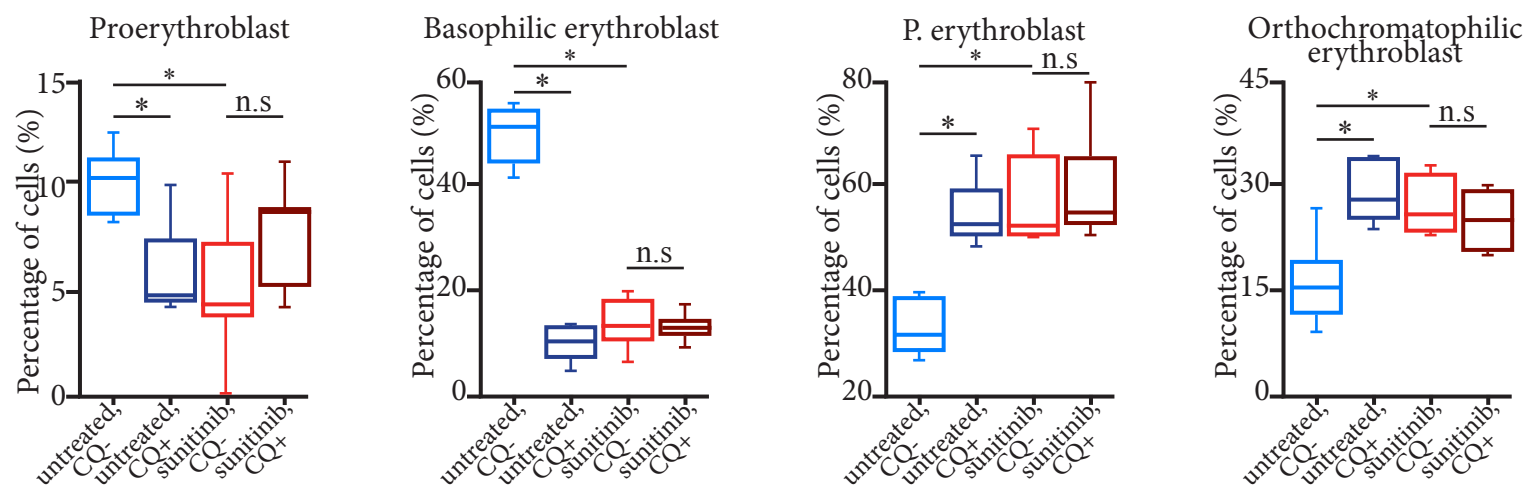

Supplement: Supplementary file 8 — Supplementary material 8. [file 40164_2025_640_MOESM8_ESM.pdf]

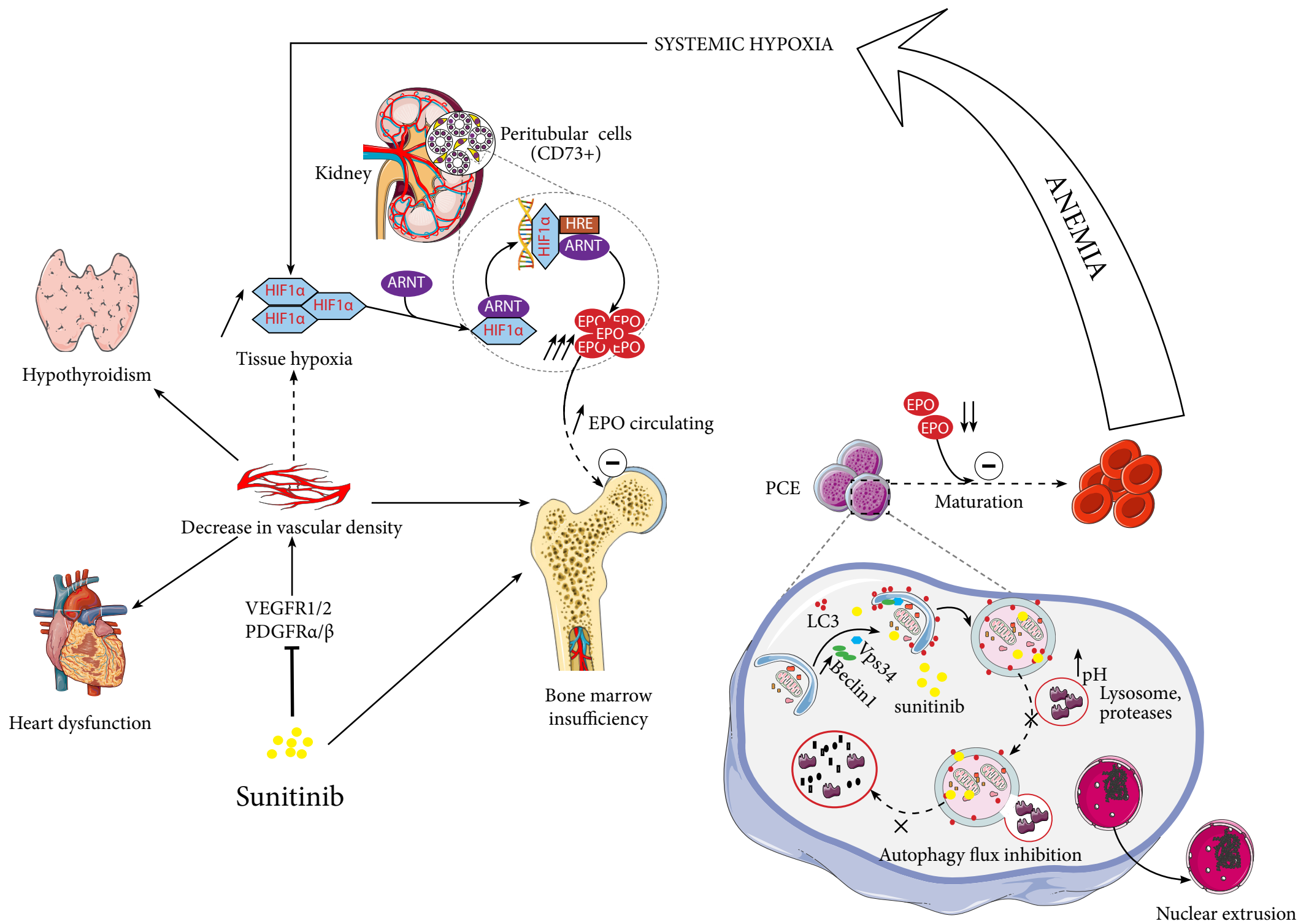

Supplement: Supplementary file 9 — Supplementary material 9. [file 40164_2025_640_MOESM9_ESM.pdf]
