# Supplementary material for: Anti-angiogenic tyrosine kinase inhibitors and the pathophysiology of their toxic effects: revisiting the treatment of anemia in metastatic cancers
Source: Exp Hematol Oncol. 2025 Apr 19;14:59. doi: 10.1186/s40164-025-00640-9 (PMC12008949; doi:10.1186/s40164-025-00640-9)
Supplement: Supplementary file 10 — Supplementary material 10. [file 40164_2025_640_MOESM10_ESM.docx]

**Supplementary Figure legends:**

**Supp.Fig1:** Diagrammatic representation of anti-angiogenic TKIs for the treatment of cancers according to haematological toxicities in a meta-analysis on 16,877 patients: A) all grades and B) grades 3-4 toxicities.

**Supp.Fig2:** Toxicities induced by anti-angiogenic TKIs are linked to a direct cytotoxic effect on normal endothelial cells.

A) The left panel shows H&E staining of thyroid sections with a significant decrease in the percentage of colloid surface area under sunitinib treatment. The right panel shows CD31 immunostaining of thyroid sections with a significant decrease in number of CD31-expressing normal vessels in sunitinib-treated mice compared to untreated mice. B) The left panel shows cardiac toxicity with a significant decrease in the left ventricular ejection fraction visualised on echocardiography, and a signifciant increase in cardiac mRNA expression of *Bnp.* The right panel shows CD31 immunostaining of heart sections with a significant decrease in number of CD31-expressing normal vessels in sunitinib-treated mice compared to untreated mice.

*: *P* < 0.05; **: *P* < 0.01; n.s: not significant. RQ: Relative quantification.

**Supp.Fig3:** Diagrammatic representation of hematopoietic stem cells and the proportion of different hematopoietic lineages using flow cytometry on bone-marrow. No significant difference is observed in living cells for LSK, HSC, MPP, thrombocytes, granulocytes, B cells, and T cells. LSK: Lin^-^Sca1^+^c-Kit^+^; LK: Lin^−^ cKit^+^; LT-HSC: Long-Term Hematopoietic Stem Cell; ST-HSC: Short-Term Hematopoietic Stem Cell; MPP: Multipotential Progenitor; MEP: Megakaryocyte–erythroid progenitor; CLP: Common Lymphoid Progenitor; preGMP: granulocyte-monocyte precursor; GMP: granulocyte-monocyte progenitor; EryP: erythroid progenitor; MKP: megakaryocyte progenitor. n.s: not significant.

**Supp.Fig4:** Characterization of the different hematopoietic lineages under anti-angiogenic TKIs using flow cytometry on bone-marrow.

The left panel provides a diagrammatic representation of the production of mature blood cells from hematopoietic stem cells. The right panel shows the proprotions of different hematopoietic lineages using flow cytometry with a nonsignificant difference in living cells for the PreGMP, MKP and GMP progenitors under sunitinib and pazopanib treatment. LSK: Lin^-^Sca1^+^c-Kit^+^; LK: Lin^−^ cKit^+^; LT-HSC: Long-Term Hematopoietic Stem Cell; ST-HSC: Short-Term Hematopoietic Stem Cell; MPP: Multipotential Progenitor; MEP: Megakaryocyte–erythroid progenitor; CLP: Common Lymphoid Progenitor; preGMP: granulocyte monocyte precursor; GMP: granulocyte-monocyte progenitor; EryP: erythroid progenitor; MKP: megakaryocyte progenitor. *: *P* < 0.05; **: *P* < 0.01; n.s: not significant.

**Supp.Fig5:** Reticulocyte count shows a significant increase in immature reticulocyte fractions in sunitinib-treated mice compared to untreated mice. *: *P* < 0.05; n.s: not significant.

**Supp.Fig6:** Sunitinib autofluorescence in erythroid colonies from untreated and sunitinib-treated mice (yellow, red arrows).

**Supp.Fig7:** Sunitinib-induced anemia was linked to autophagy flux inhibition in erythroid progenitors.

A) The left panel shows mRNA expression levels of *Bnip3l, Becn1* in erythroid colonies from sunitinib-treated and untreated mice. The right panel shows mRNA expression levels of *Lc3* in erythroid colonies with or without exposure to hydrocloroquine (CQ). B) Counts of erythroid progenitors in erythroid colonies using transmission electron microscopy.

P: Proerythroblast; B: Basophilic erythroblast; PE: polychromatophilic erythroblast; O: orthochromatophilic erythroblast.

CQ: hydrocloroquine; *: *P* < 0.05; **: *P* < 0.01; n.s: not significant.

**Supp.Fig8:** Diagrammatic representation of the pathophysiology of anti-angiogenic TKI-induced anemia. PCE: Polychromatic erythrocyte; ARNT: Aryl Hydrocarbon Receptor Nuclear Translocator, HIF: Hypoxia-inducible Factor; EPO: Erythropoietin; GFR, Vascular endothelial growth factor receptor ; PDGFRβ, Platelet-derived growth factor receptor β; HRE: Hypoxia-Responsive Elements.
